# Chemical Composition and Biological Activities of *Lagopsis supina* Extract: Antioxidant, Adipogenic, and Ani-Inflammatory Effects

**DOI:** 10.3390/ph18020150

**Published:** 2025-01-23

**Authors:** Juhyun Choi, Duc Dat Le, Nayoung Roh, Jiseok Lee, Deumaya Shrestha, Thientam Dinh, Vinhquang Truong, Badamtsetseg Bazarragchaa, Soo-Yong Kim, Sung-Suk Suh, Mina Lee, Jong Bae Seo

**Affiliations:** 1 Department of Biomedicine, Health & Life Convergence Sciences, BK21 Four, Biomedical and Healthcare Research Institute, Mokpo National University, Muan 58554, Jeonnam, Republic of Korea; cjh000126@gmail.com (J.C.); youngna5201@naver.com (N.R.); leejisuk722@naver.com (J.L.); sungsuksuh@mokpo.ac.kr (S.-S.S.); 2 College of Pharmacy and Research Institute of Life and Pharmaceutical Sciences, Sunchon National University, 255 Jungangno, Suncheon 57922, Jeonnam, Republic of Korea; ddle@scnu.ac.kr (D.D.L.); thientamm.2001@gmail.com (T.D.); quangvtruong00@gmail.com (V.T.); 3 Department of Biosciences, Mokpo National University, Muan 58554, Jeonnam, Republic of Korea; sitamon2018@gmail.com; 4 Natural History Museum of Mongolia, Ulaanbaatar 15141, Mongolia; batamtsetseg71@gmail.com; 5 International Biological Material Research Center, Korea Research Institute of Bioscience and Biotechnology, Daejeon 34141, Republic of Korea; soodole@kribb.re.kr; 6 Department of Natural Cosmetics Science and Natural Cosmetics Research Institute, Sunchon National University, 255 Jungangno, Suncheon 57922, Jeonnam, Republic of Korea

**Keywords:** *Lagopsis supina*, antioxidant, adipogenic transcription, UHPLC-MS/MS, inflammation

## Abstract

Background/Objectives: *Lagopsis supina*, a traditional Chinese medicine valued for its diuretic properties, has limited research on its antioxidant, adipogenic, and anti-inflammatory effects. This study aimed to investigate the chemical composition and biological activities of *Lagopsis supina* extract (LSE). Methods: LSE was prepared and evaluated for antioxidant activity, effects on adipocyte differentiation in 3T3-L1 preadipocytes, and anti-inflammatory properties in RAW 264.7 macrophages. Ultra-high-performance liquid chromatography-electrospray ionization Orbitrap tandem mass spectrometry (UHPLC-ESI-Orbitrap-MS/MS)-based molecular networking was used to characterize its secondary metabolites. Results: LSE exhibited antioxidant activity in DPPH and ABTS assays. It significantly enhanced the differentiation of 3T3-L1 preadipocytes into mature adipocytes during early and intermediate stages by upregulating adipogenic transcription factors such as PPARγ, C/EBPα, and C/EBPβ, along with promoting cyclin E expression. LSE also increased PPARγ activity and the expression of its target genes, such as Glut 4, PEPCK, FABP4, and Plin2. Moreover, LSE inhibited lipopolysaccharide (LPS)-induced inflammation in RAW 264.7 macrophages by downregulating pro-inflammatory mediators (iNOS, COX-2, TNF-α, IL-6) and inhibiting extracellular signal-regulated kinase (ERK) phosphorylation. Chemical profiling revealed eight major compound groups: glycosides, organic acids, terpenoids, flavonoids, phenylglycosides, phenolics, fatty acids, and others characterized by their mass fragmentation patterns, precursors, and UV absorption spectra. In silico analysis confirmed these compounds’ bioactivities, demonstrating strong interactions and binding affinities with antioxidant, adipogenic, and anti-inflammatory protein targets. Conclusions: These findings highlight LSE’s triple therapeutic potential: antioxidant activity, adipogenesis promotion, and inflammation attenuation. LSE emerges as a promising therapeutic candidate for managing obesity and related inflammatory complications.

## 1. Introduction

Obesity, a global public health challenge, is associated with an increased risk of numerous chronic diseases and health complications, either as an independent condition or in combination with other factors [1]. According to the World Health Organization (WHO), 39% of adults (1.9 billion) worldwide were classified as overweight in 2016, of whom 13% (over 650 million) were obese. Alarmingly, obesity also affected 340 million children and adolescents aged 5–19 years and 24 million children under 5 years [2]. In 2019, a nationwide cross-sectional study in China revealed that nearly half the population was overweight or obese, with this prevalence directly linked to a surge in complications such as fatty liver disease, prediabetes, dyslipidemia, and hypertension [3]. Obesity notably amplifies the risk of morbidity and mortality, especially from cardiovascular disease (CVD) and diabetes, while contributing to a spectrum of chronic conditions, including osteoarthritis, liver and kidney disease, sleep apnea, and depression [4].

A key factor of obesity-related health issues is chronic, low-grade inflammation, which arises from adipose tissue expansion and disrupted insulin/mTORC2 signaling pathways. This inflammatory process underpins insulin resistance, pancreatic beta-cell dysfunction, and systemic metabolic dysregulation [5]. The inflammatory cascade begins with the recruitment of monocytes and the activation of pro-inflammatory macrophages, which secrete cytokines such as tumor necrosis factor-alpha (TNF-α) and interleukin-6 (IL-6). These cytokines amplify inflammation [6], perpetuating a cycle that exacerbates metabolic complications like type 2 diabetes [7].

Lipopolysaccharides (LPSs), commonly employed in vitro to simulate inflammatory responses, activate macrophages via Toll-like receptor 4 (TLR4) and MD-2 complexes. This activation triggers signaling through the mitogen-activated protein kinase (MAPK) pathways, including extracellular signal-regulated kinase (ERK), c-Jun N-terminal kinase, and p38. These pathways regulate the expression of critical inflammatory mediators such as inducible nitric oxide synthase (iNOS), cyclooxygenase-2 (COX-2), and cytokines like TNF-α and IL-6 [8]. Nitric oxide (NO), produced by iNOS, serves as both a marker and a contributor to the pathophysiology of obesity-driven inflammation [9]. Innovative therapeutic strategies that target MAPK phosphorylation, iNOS, and COX-2 within macrophages represent promising avenues for reducing the inflammatory burden of obesity and its associated complications [10].

*Lagopsis supina* (Steph.) Ik.-Gal., a traditional Chinese medicine predominantly found in Northern China, including Ningxia, Shanxi, Gansu, Henan, and Shaanxi provinces, has long been used to treat ailments such as oligomenorrhea, hemiplegia, blood stagnation, amenorrhea, anemic dizziness, and inflammation and as a diuretic [11,12,13,14,15]. Recent research reveals that *L*. *supina* extract (LSE) mitigates myocardial infarction damage in vivo by promoting angiogenesis, reducing oxidative stress, and modulating inflammation through pathways such as VEGF and HMGB1 [16]. LSE comprises over 80 identified compounds, including diterpenoids, phenylethanoid glycosides, and flavonoid glycosides [11,17,18,19]. While studies have documented its diuretic [17,19], anti-inflammatory [12,20], and anti-neuroinflammatory properties [21], its influence on adipocyte differentiation and macrophage-driven inflammation remains unexamined.

This study explored the therapeutic potential of LSE in managing oxidative stress and obesity-related inflammation. Using in vitro models, we evaluated its radical scavenging activity, impact on adipocyte differentiation in 3T3-L1 cells, and anti-inflammatory effects in LPS-stimulated RAW 264.7 macrophages. LSE was found to enhance adipogenesis and lipogenesis in 3T3-L1 cells while significantly inhibiting LPS-induced nitric oxide (NO) production in RAW 264.7 macrophages, likely through the modulation of MAPK phosphorylation, iNOS, and COX-2. Moreover, a UHPLC-ESI-Orbitrap-MS/MS-based molecular networking approach, integrated with open-platform-guided isolation and dereplication, enabled the identification of bioactive metabolites in LSE. Molecular docking further highlighted the strong binding affinities of key components to target proteins involved in adipogenesis and inflammation. These findings suggest that LSE has significant potential as a natural therapeutic agent for addressing the inflammatory underpinnings of obesity and its associated metabolic disorders.

## 2. Results

### 2.1. Antioxidative Capacity of LSE Through Radical Scavenging Activity

**Figure 1:**
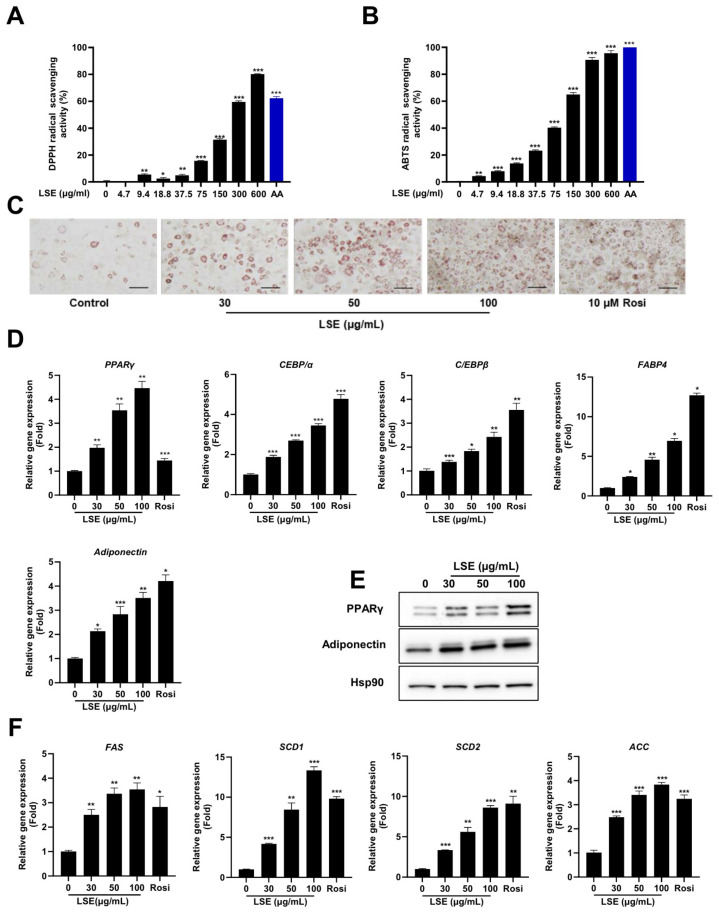
Antioxidant activities of *Lagopsis supina* extract (LSE): The radical scavenging activity of DPPH (**A**) and ABTS (**B**) was assessed at varying concentrations of LSE. Results are presented as mean ± SEM (*n* = 3). Ascorbic acid (AA, 150 µg/mL) served as positive control. Adipogenesis and lipogenic gene expression induced by LSE: Effects of LSE on adipogenesis were studied in 3T3-L1 cells differentiated using induction medium containing 0.17 × IBMX, dexamethasone, and insulin (DMI). Cells were treated with LSE (30, 50, 100 µg/mL), a control treatment, or rosiglitazone (10 µM) as a positive control. (**C**) Oil red O staining visualized intracellular lipid accumulation; the scale bar represents 100 µm at 200× magnification. (**D**) Quantitative real-time PCR (qRT-PCR) analyzed adipogenesis-related gene expression, including PPARγ, C/EBPα, C/EBPβ, FABP4, and adiponectin. (**E**) Western blot analysis evaluated protein levels of PPARγ and adiponectin, with HSP90 used as a loading control. (**F**) qRT-PCR quantified mRNA levels of lipogenesis-associated genes such as SCD1, SCD2, FAS, and ACC. Data are shown as mean ± SEM from two independent experiments (*n* = 4). Statistical significance relative to the control group is denoted as * *p* < 0.05, ** *p* < 0.01, and *** *p* < 0.001.

To evaluate the antioxidant capacity of LSE, DPPH and ABTS assays were conducted to measure free radical scavenging activities. The scavenging activity was expressed as the percentage inhibition of DPPH and ABTS radicals. Figure 1A,B depicts the average scavenging percentages for DPPH and ABTS at varying LSE concentrations. The findings indicate that LSE’s radical scavenging activity increases with concentration. At 150 µg/mL, LSE demonstrated a moderate inhibition of 31.44% ± 0.73% compared to ascorbic acid (AA), the standard antioxidant, which achieved a significant DPPH inhibition of 62.26% ± 1.20%. Similarly, for ABTS, LSE showed an inhibition of 65.03% ± 1.40% at 150 µg/mL, while AA exhibited a potent inhibitory activity of 99.36% ± 0.02%. The IC_50_ values derived from the DPPH and ABTS assays support these results, with LSE showing higher IC_50_ values (234.5 μg/mL for DPPH and 109.4 μg/mL for ABTS), indicative of lower antioxidant potential. Compared to synthetic AA, LSE exhibits moderate antioxidant activity.

### 2.2. LSE Stimulates 3T3-L1 Adipocyte Differentiation Without Cytotoxicity

To assess the cytotoxicity of LSE in 3T3-L1 preadipocytes, a WST-8 cell viability assay was performed. As shown in Figure S1A, treatment with 10–200 μg/mL LSE for 24 h did not significantly affect cell viability, indicating that LSE is non-toxic to 3T3-L1 preadipocytes under these conditions. Adipocyte differentiation was evaluated using induction media containing varying concentrations of a standard differentiation cocktail: 3-isobutyl-1-methylxanthine, dexamethasone, and insulin (MDI). Post-confluent 3T3-L1 preadipocytes were treated with 0.17×, 0.33×, or 1× MDI during adipogenesis. Oil red O staining revealed a concentration-dependent increase in intracellular lipid accumulation (Figure S1B). Additionally, the expression of adipocyte-specific genes, including PPARγ, FABP4, and adiponectin, was significantly elevated in cells treated with higher MDI concentrations, consistent with the lipid staining results (Figure S1C). Differentiated adipocytes in the 0.17 × MDI-treated group represented about 25% of those in the 1 × MDI-treated group. Based on these findings, 0.17 × MDI was selected as the induction medium for subsequent experiments on LSE’s effect on adipocyte differentiation.

To investigate LSE’s impact on adipogenesis, 3T3-L1 preadipocytes were treated with LSE at 30, 50, and 100 μg/mL in differentiation media. Oil red O staining showed a significant, dose-dependent increase in lipid accumulation in LSE-treated cells compared to untreated controls (Figure 1C). These results suggest LSE promotes preadipocyte maturation into adipocytes without inducing cytotoxicity.

To elucidate the underlying mechanisms, qRT-PCR analysis assessed adipogenic gene expression. LSE treatment induced a dose-dependent upregulation of PPARγ, C/EBPα, C/EBPβ, adiponectin, and FABP4 compared to controls (Figure 1D). Western blot analysis confirmed these results, showing increased protein expression of PPARγ and adiponectin in a dose-dependent manner (Figure 1E). LSE treatment also significantly upregulated lipogenic genes, including FAS, SCD-1, SCD-2, and ACC (Figure 1F). These findings collectively demonstrate that LSE promotes 3T3-L1 preadipocyte differentiation into mature adipocytes by upregulating genes associated with adipogenesis and lipogenesis.

### 2.3. LSE Exhibits Enhanced Effects on Adipocyte Differentiation During Early and Intermediate Stages

**Figure 2:**
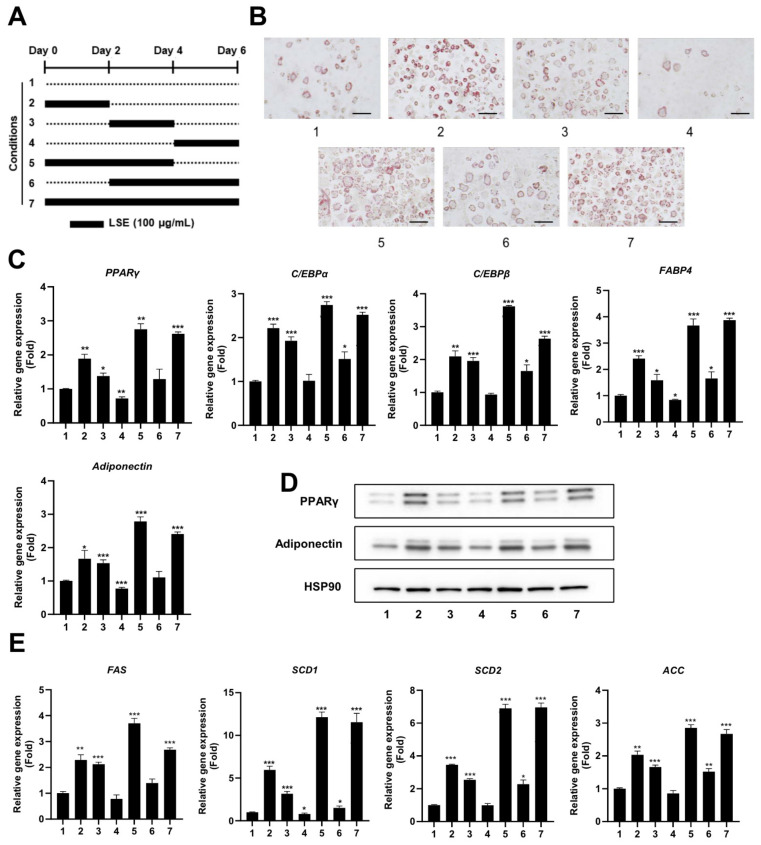
Time-dependent effects of LSE on adipocyte differentiation: (**A**) A schematic diagram illustrates the experimental design, where 3T3-L1 cells were treated with 100 µg/mL LSE for different durations (Groups 1–7), starting 2 days after reaching confluence. (**B**) Oil red O staining visualized intracellular lipid accumulation after 6 days of treatment. The scale bar represents 100 µm at 200× magnification. (**C**) On Day 6, total RNA was extracted, and qRT-PCR analyzed mRNA levels of adipogenesis-related genes, including PPARγ, C/EBPα, C/EBPβ, FABP4, and Adiponectin. (**D**) Western blotting evaluated protein levels of PPARγ and Adiponectin, using HSP90 as a loading control. (**E**) qRT-PCR quantified mRNA expression of lipogenesis-related genes, including SCD1, SCD2, FAS, and ACC. Data are presented as mean ± SEM from two independent experiments. Statistical significance relative to the control group is denoted as * *p* < 0.05, ** *p* < 0.01, and *** *p* < 0.001.

To pinpoint the specific stage of adipocyte differentiation most influenced by LSE, preadipocytes were treated with LSE at different time points during the differentiation process (Figure 2A). As shown in Figure 2B, Oil red O staining revealed that treatment during the early stages (conditions 2, 5, and 7) resulted in the most pronounced lipid accumulation in adipocytes, followed by treatment during the intermediate stages (conditions 3 and 6). In contrast, treatment during the late stage (condition 4) had the least effect compared to the untreated control group (condition 1). qRT-PCR analysis showed a significant upregulation of adipogenic markers, including PPARγ, C/EBPα, C/EBPβ, FABP4, and adiponectin, in all treatment groups relative to controls, with the most substantial increase observed in the early and intermediate treatment groups (Figure 2C). Western blot analysis confirmed these findings, showing elevated protein levels of PPARγ and adiponectin in early and intermediate treatment groups, with minimal changes in the late-stage group (Figure 2D). Similarly, lipogenic gene expressions, including SCD1, SCD2, FAS, and ACC, were significantly upregulated in early and intermediate treatment groups (Figure 2E). These results indicate that LSE exerts its strongest influence on adipocyte differentiation when applied during the early and intermediate stages of the process.

### 2.4. LSE Promotes Adipocyte Differentiation Through Enhanced Expression of Key Regulatory Genes

**Figure 3:**
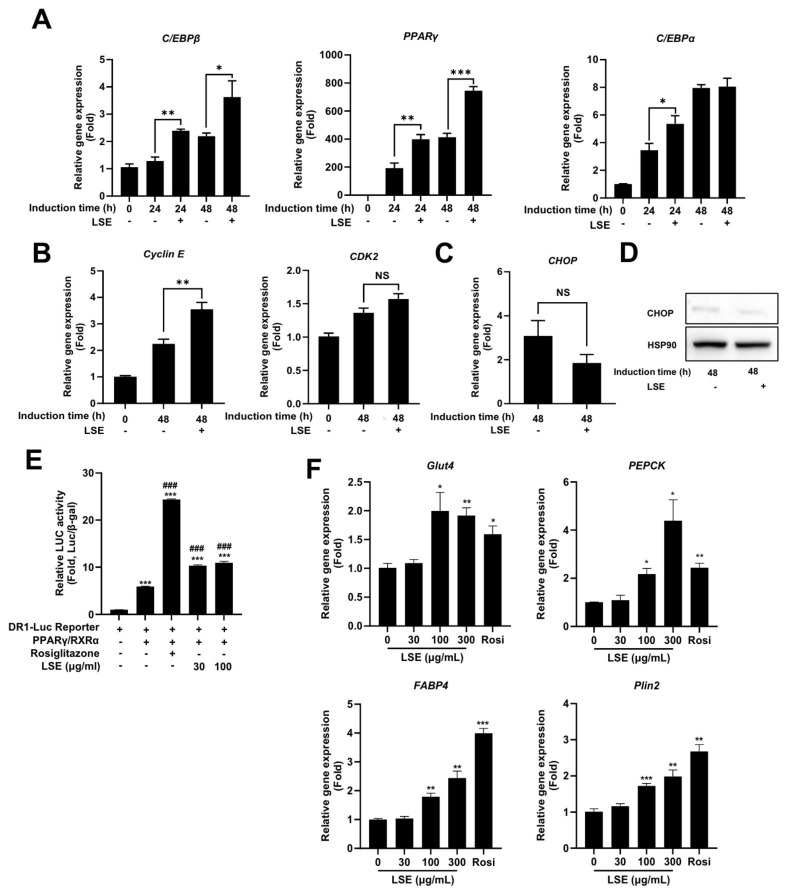
Effects of LSE on early adipogenesis and cell cycle regulation: 3T3-L1 cells were cultured to confluence over 48 h, followed by treatment with 100 μg/mL LSE. (**A**) qRT-PCR analyzed mRNA levels of adipogenesis-related genes, including PPARγ, C/EBPα, and C/EBPβ. (**B**) mRNA levels of cell cycle-related genes Cyclin E and CDK2 were quantified using qRT-PCR. (**C**) CHOP mRNA levels were assessed via qRT-PCR, and (**D**) protein expression of CHOP, a cellular stress response marker, was analyzed by Western blotting. (**E**) LSE’s effect on PPARγ activity was determined by transfecting h293a cells with DR-1-Luc containing PPRE sequences upstream of a luciferase gene; cells were treated with LSE (30 and 100 μg/mL) or rosiglitazone (10 μM) for 24 h, and luminescence levels were normalized to β-gal assay O.D. values. (**F**) The effect of LSE on PPARγ target gene expression was examined by treating differentiated adipocytes with 100 μg/mL LSE, 10 μM rosiglitazone (positive control), or DMSO (negative control) for 2 days, followed by qRT-PCR analysis. Data represent mean ± SEM from two independent experiments. Statistical significance is denoted as * *p* < 0.05, ** *p* < 0.01, and *** *p* < 0.001; ^###^ *p* < 0.001 compared to DR-Luc Reporter/PPARγ/RXRα -transfected cells; NS = not significant.

To explore the mechanisms underlying LSE-induced adipocyte differentiation, we examined the expression of key regulatory genes. LSE treatment during the initial differentiation stages (Day 0–2) significantly increased mRNA levels of C/EBPβ, PPARγ, and C/EBPα, essential transcription factors for adipocyte formation (Figure 3A).

Additionally, LSE treatment upregulated cyclin E mRNA expression, a protein critical for mitotic clonal expansion (MCE), a prerequisite for preadipocyte differentiation (Figure 3B). This suggests that LSE not only activates adipogenic gene transcription but also facilitates preadipocyte proliferation by enhancing cell cycle progression. Notably, the expression of CHOP, a negative regulator of adipocyte differentiation, remained unchanged at both mRNA and protein levels following LSE treatment (Figure 3C,D), suggesting that LSE promotes differentiation through a CHOP-independent pathway. Collectively, these findings demonstrate that LSE enhances adipocyte development by promoting the expression of key regulatory genes and supporting cell division processes critical for differentiation.

### 2.5. LSE Stimulates PPARγ Activity and Upregulates PPARγ Target Genes

PPARγ is a critical transcription factor in adipocyte differentiation. To assess whether LSE modulates PPARγ activity, a luciferase reporter assay was performed using a PPRE-containing reporter construct that binds the PPARγ/RXRα heterodimer. As shown in Figure 3E, rosiglitazone, a known PPARγ agonist, significantly enhanced luciferase activity, serving as a positive control. Similarly, LSE increased luciferase activity in a concentration-dependent manner, demonstrating its ability to directly regulate PPARγ activity. To further evaluate LSE’s effects on PPARγ-mediated gene expression, the expression levels of key target genes, including glucose transporter 4 (Glut4), phosphoenolpyruvate carboxykinase (PEPCK), fatty acid-binding protein 4 (FABP4), and perilipin-2 (Plin2), were evaluated in differentiated adipocytes following LSE treatment (Figure 3F). LSE significantly upregulated these target genes, reinforcing its role in activating PPAR γ and promoting adipocyte differentiation.

### 2.6. Cytotoxicity and Nitric Oxide (NO) Inhibitory Activity of LSE in RAW264.7 Macrophages

**Figure 4:**
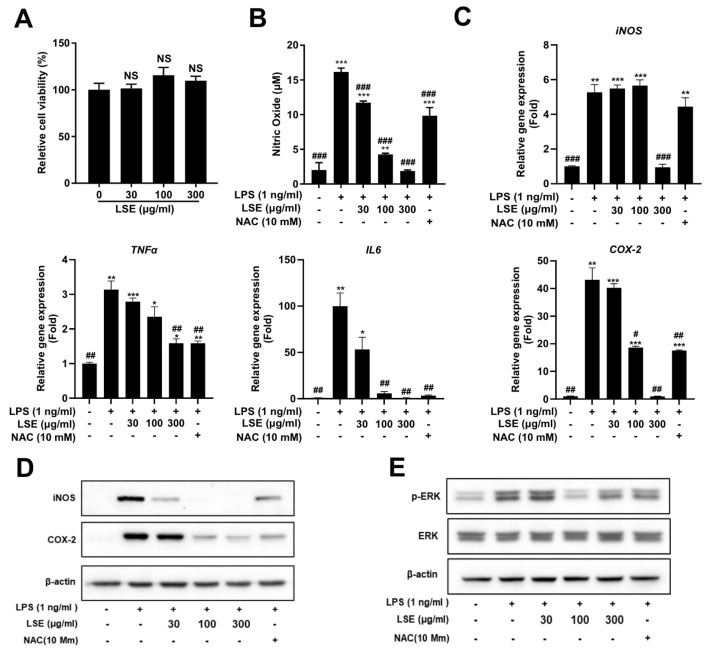
Effects of *Lagopsis supina* extract (LSE) on RAW 264.7 cell viability and lipopolysaccharide (LPS)-induced inflammation: (**A**) RAW 264.7 macrophages were treated with varying concentrations of LSE for 24 h, and cell viability was assessed using a WST-8 assay. Results are expressed as a percentage relative to the control group (*n* = 5). (**B**) RAW 264.7 macrophages were pre-treated with LSE for 2 h, followed by LPS stimulation for 24 h. Nitric oxide (NO) production was quantified using the Griess reaction assay, with N-acetylcysteine (NAC) as a positive control. Data represents mean ± SEM from two independent experiments (*n* = 4). “ns” denotes no significant difference from the control group. (**C**) qRT-PCR analyzed mRNA levels of inflammatory genes, including iNOS, COX-2, TNF-α, and IL-6, with NAC serving as a positive control. (**D**) Western blot analysis evaluated protein levels of iNOS and COX-2, with NAC as a positive control. Data represent mean ± SEM from two independent experiments (*n* = 4 for each condition). (**E**) RAW 264.7 macrophages were pre-treated with LSE (30, 100, and 300 μg/mL) for 2 h, followed by LPS stimulation for 24 h. Western blot analysis examined the expression of ERK and its phosphorylated form (p-ERK). NAC was used as positive control. ERK denotes extracellular signal-regulated kinase, and p-ERK refers to its phosphorylated form. Statistical significance is indicated as follows: *** *p* < 0.001, ** *p* < 0.01, * *p* < 0.05 compared to unstimulated control cells; ^#^ *p* < 0.05, ^##^ *p* < 0.01, ^###^ *p* < 0.001 compared to LPS-stimulated cells; NS = not significant.

To assess the cytotoxicity of LSE, RAW264.7 cells were treated with concentrations ranging from 30 to 300 µg/mL. Cell viability assays confirmed that LSE was non-toxic, even at the highest concentration (300 µg/mL) (Figure 4A). The anti-inflammatory properties of LSE were then evaluated by measuring NO production in LPS-stimulated RAW264.7 cells. LSE treatment reduced NO production in a dose-dependent manner, with the highest concentration (300 µg/mL) exhibiting a stronger inhibitory effect than the anti-inflammatory agent N-acetylcysteine (NAC) (Figure 4B). These findings establish LSE as a safe and potent modulator of inflammatory responses in macrophages.

### 2.7. Molecular Mechanisms Underlying the Anti-Inflammatory Effect of LSE

To investigate the molecular mechanisms underlying LSE’s anti-inflammatory effects, we analyzed the expression of key inflammatory mediators and the involvement of specific signaling pathways. qRT-PCR analysis showed that LSE treatment significantly downregulated pro-inflammatory genes, including iNOS, COX-2, TNF-α, and IL-6, in a dose-dependent manner in LPS-stimulated RAW264.7 macrophages (Figure 4C). Western blot analysis confirmed these findings, revealing reduced protein levels of iNOS and COX-2 after LSE treatment, with effects more pronounced than those of the anti-inflammatory agent NAC (Figure 4D).

To investigate the molecular basis of LSE’s anti-inflammatory effects, its impact on the ERK signaling pathway, a critical regulator of inflammation, was assessed. Western blot analysis revealed that LSE pretreatment significantly inhibited LPS-induced phosphorylation of ERK (p-ERK) in RAW264.7 macrophages (Figure 4E). Notably, NAC did not alter p-ERK levels, indicating that LSE specifically suppresses inflammatory responses by attenuating ERK phosphorylation. These findings suggest that LSE exerts its anti-inflammatory effects through a dual mechanism: the downregulation of pro-inflammatory mediators and the inhibition of ERK signaling.

### 2.8. Identification of Metabolites from LSE

**Figure 5:**
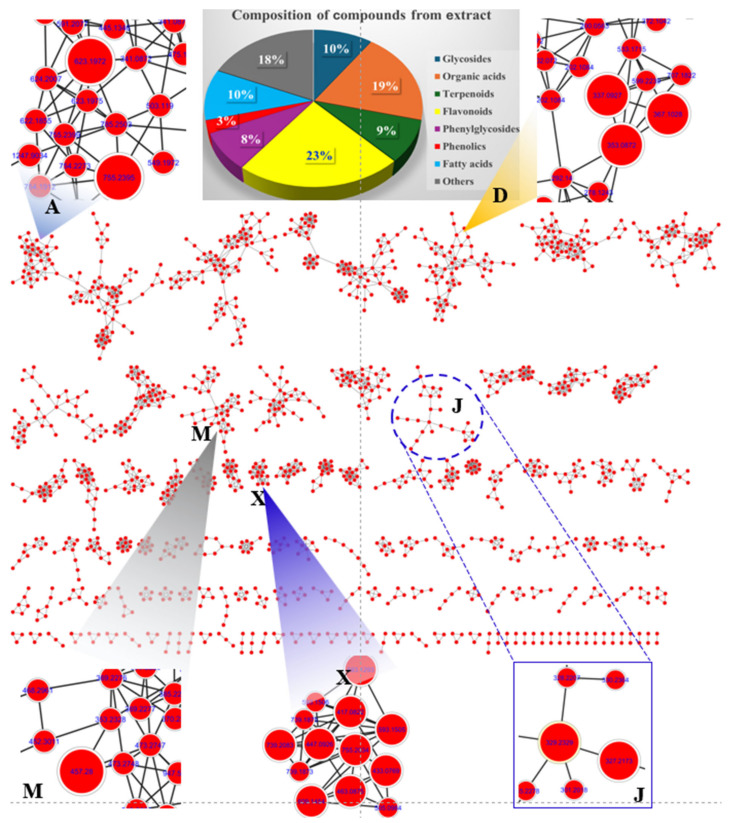
Feature-based molecular network of LSE and specific clusters (A, D, M, J, and X).

The chemical profile of LSE was analyzed using UHPLC-Orbitrap-MS/MS, generating ion chromatograms in negative mode (Figure S2, Supplementary Materials). Peaks were well separated with precursor masses reaching approximately 1500 Da. To identify secondary metabolites (Table 1), data from the UHPLC-Orbitrap-MS/MS system analysis underwent support by GNPS open web tools for feature-based molecular networking visualization (Figure 5). Nodes in the molecular network were clustered based on similar isotope patterns of precursors.

Cluster A highlighted the presence of two major phenylglycosides: [(2*R*,3*R*,4*R*,5*R*,6*R*)-2-[[(2*R*,3*R*,4*R*)-3,4-dihydroxy-4-(hydroxymethyl) oxolan-2-yl]oxymethyl-4-[(2*S*,3*R*,4*R*,5*R*,6*S*)-4,5-dihydroxy-6-methyl-3-[(2*S*,3*R*,4*S*,5*S*)-3,4,5-trihydroxyoxan-2-yl]oxyoxan-2-yloxy-6-[2-(3,4-dihydroxyphenyl)ethoxy]-5-hydroxyoxan-3-yl] (*E*)-3-(3,4-dihydroxyphenyl)prop-2-enoate and acteoside. These compounds, observed at *m*/*z* 755.2393 [M-H]^−^ and *m*/*z* 623.1972 [M-H]^−^, respectively, were identified as major constituents of *L*. *supina* [22]. Cluster D revealed nodes corresponding to quinic acid derivatives with specific fragments at *m*/*z* 191 [M-H]^−^. These included precursor ion peaks at *m*/*z* 367.1028 [M-H]^−^, 353.0872 [M-H], and 337.0927 [M-H]^−^, indicating the presence of organic acids such as 3-*O*-feruloylquinic acid, neochlorogenic acid, and coumaroyl quinic acid, respectively. Proposed biosynthetic pathways suggest that 3-*O*-feruloylquinic acid undergoes demethylation to form neochlorogenic acid via methyl group reduction. Neochlorogenic acid, in turn, may lose a water molecule to produce coumaroyl quinic acid [23]. Cluster J displayed nodes with precursor ion peaks at *m*/*z* 327.2173 [M-H]^−^ and at *m*/*z* 329.2329 [M-H]^−^, assigned to fatty acids identified as (*Z*)-9,12,13-trihydroxyoctadec-15-enoic acid and 2-(2-acetyloxy-12-hydroxytridecyl)-4,6-dihydroxybenzoic acid, respectively [22]. It is proposed that these compounds may interconvert by exchanging two hydrogen atoms during biosynthetic processing. Cluster M showed a major peak at *m*/*z* 457.2798 [M-H], corresponding to [4,5-dihydroxy-3,4-bis(hydroxymethyl)-4a,8,8-trimethyl-5,6,7,8a-tetrahydro-1H-naphthalen-1-yl]octanoate. Cluster X displayed nodes associated with flavonoid glycosides, categorized into kaempferol and quercetin backbones. The peaks detected at *t*_R_ of 8.808, 9.596, 10.825, 11.842, and 14.111 min shared a mass fragment at *m*/*z* 284 [M-H]^−^, corresponding to the kaempferol backbone. Their precursor ions were observed at *m*/*z* 739.2083, 593.1505, 447.0926, 417.0822, and 593.1293 [M-H]^−^, identified as 3-[4,5-dihydroxy-3-[(2*R*,3*R*,4*R*,5*R*,6*S*)-3,4,5-trihydroxy-6-methyloxan-2-yl].oxy-6-[[(2*R*,3*R*,4*R*,5*R*,6*S*)-3,4,5-trihydroxy-6-methyloxan-2-yl]oxymethyl]oxan-2-yl]oxy-5,7-dihydroxy-2-(4-hydroxyphenyl)chromen-4-one; kaempferol 3-*O*-neohesperidoside; kaempferol-3-*O*-glucoside; kaempferol-3-*O*-arabinoside; and tribuloside, respectively. The biosynthetic pathway suggests that these secondary metabolites undergo deglycosylation to reduce molecular weight, converting the peak at *m*/*z* 739.2083 [3-[4,5-dihydroxy-3-[(2R,3R,4R,5R,6S)-3,4,5-trihydroxy-6-methyloxan-2-yl]oxy-6-[[(2R,3R,4R,5R,6S)-3,4,5-trihydroxy-6-methyloxan-2-yl]oxymethyl]oxan-2-yl]oxy-5,7-dihydroxy-2-(4-hydroxyphenyl)chromen-4-one] to *m*/*z* 593.1505 [kaempferol 3-*O*-neohesperidoside] by removing a rhamnosyl molecule.

A fragmentation pathway was similarly observed for the peak at *m*/*z* 593.1505 [kaempferol 3-*O*-neohesperidoside], which transitions to the peak at *m*/*z* 447.0926 through the loss of a rhamnosyl molecule, forming kaempferol-3-*O*-glucoside. Subsequently, the peak at *m*/*z* 447.0926 reduces a methylene-hydroxy group to produce the peak at *m*/*z* 417.0822, corresponding to kaempferol-3-*O*-arabinoside [24]. The peaks detected at *t*_R_ of 8.213, 9.405, 9.896, and 10.774 min showed the same daughter peak at *m*/*z* 301 [M-H]^−^, indicating the quercetin backbone. It is proposed that the peak at *m*/*z* 739.2083 [3-[4,5-dihydroxy-3-[(2*R*,3*R*,4*R*,5*R*,6*S*)-3,4,5-trihydroxy-6-methyloxan-2-yl]oxy-6-[[(2*R*,3*R*,4*R*,5*R*,6*S*)-3,4,5-trihydroxy-6-methyloxan-2-yl]oxymethyl]oxan-2-yl]oxy-5,7-dihydroxy-2-(4-hydroxyphenyl) chromen-4-one] undergoes hydroxylation to produce the ion peak at *m*/*z* 755.2034 [Manghaslin]. This peak further generates the ion peak at *m*/*z* 609.1454 [M-H]^−^ via deglycosylation, forming rutin [25]. A biosynthetic transformation pathway was observed where the peak at *m*/*z* 609.1454 transitions to peaks at *m*/*z* 463.0875 and *m*/*z* 433.0769 through successive reductions of a rhamosyl molecule and a methylene-hydroxy group, respectively. The remaining peaks in the chromatograms were annotated using the MS-Dial program version 5.1.230912, comparing experimental mass data with public mass databanks. The precursor ion peaks at *m*/*z* 409.2223 [M-H]^−^ and at *m*/*z* 409.2224 [M-H]^−^ were identified as the terpenoid compounds forskolin and 1-*epi* coleonol, respectively. Another prominent terpenoid peak at *m*/*z* 393.2276 [M-H]^−^ was identified as grayanotoxin I by interpreting its mass spectrum in conjunction with mass databanks. Additional compounds were identified by dereplication of mass precursors and fragmentation patterns, alongside comparisons with previous reports, an in-house library, or public mass databanks.

To estimate the metabolite content in the extract, the analytical data were processed based on peak areas from chromatograms in negative ion mode. Peaks 55, 57, 85, 88, 91, 101, 106, 108, 109, and 113 were identified as major peaks, exhibiting higher content relative to others in the chromatogram (Figure S3, Supplementary Materials).

### 2.9. Molecular Docking Analysis

**Figure 6:**
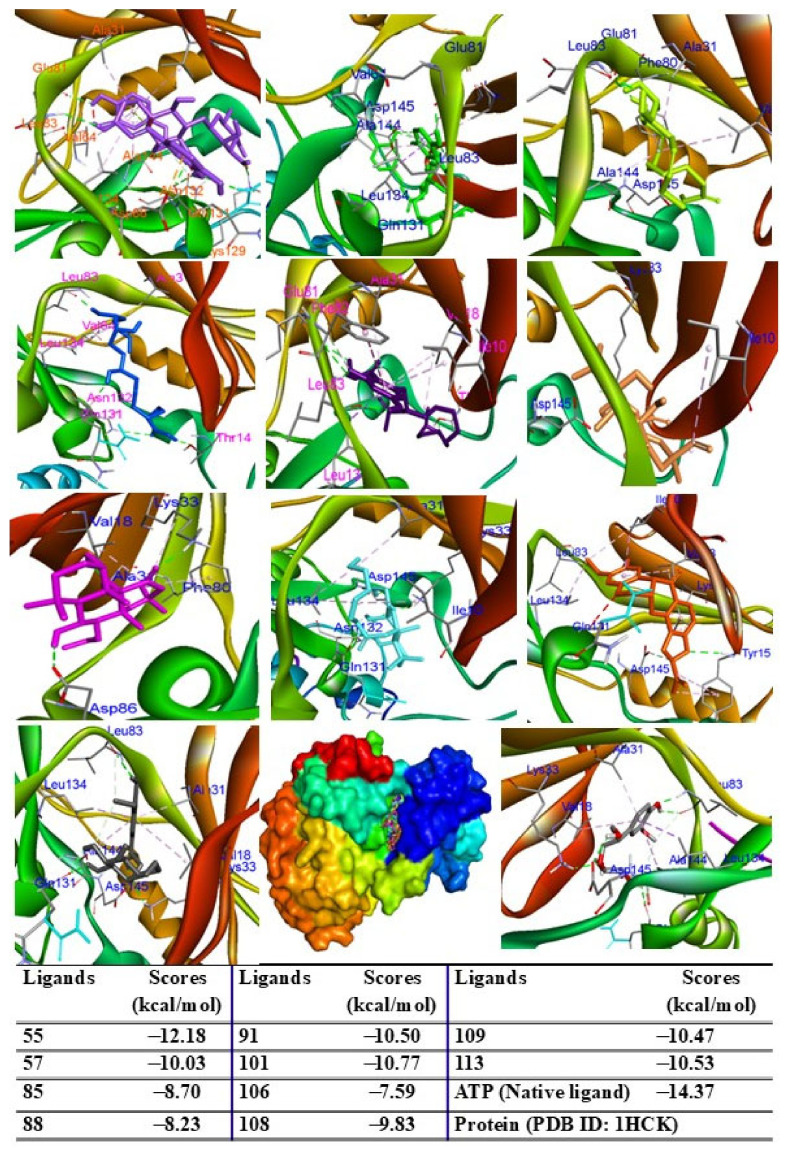
Interactions of compounds 55 (Raspberry), 57 (Green), 85 (Limon), 88 (Blue), 91 (Purple blue), 101 (Wheat), 106 (Magenta), 108 (Cyan), 109 (Orange), and 113 (Gray) with amino acid when they were docked into target protein (PDB ID 1HCK).

To evaluate the biological potential of the extract, in silico studies were conducted to predict the activity of major compounds against antioxidant, adiponectin, PPARγ, IL-6, TNF-α, iNOS, COX-2, and ERK proteins using the AutoDock4.0 program. Initially, docking protocols were validated by redocking native ligands into protein–ligand complexes. The root mean square deviation between the original and redocked ligands was calculated to be less than 2 Å, confirming the accuracy of the docking procedure. When major peaks were docked into the 1HCK protein, the ligands and redocked native ligands occupied the same binding pose within the protein, validating the reliability of the docking protocol (Figure S4, Supplementary Materials). Among the compounds tested, peak 55 exhibited the lowest binding energy (ΔG = −12.18 kcal/mol), followed by peaks 101 (ΔG = −10.77 kcal/mol), 113 (ΔG = −10.53 kcal/mol), 91 (ΔG = −10.50 kcal/mol), 109 (ΔG = −10.47 kcal/mol), 57 (ΔG = −10.03 kcal/mol), 108 (ΔG = −9.83 kcal/mol), 85 (ΔG = −8.70 kcal/mol), 88 (ΔG = −8.23 kcal/mol), and 106 (ΔG = −7.59 kcal/mol) (Figure 6). These results suggest that hydrogen bonds with key residues, such as THR14 and TYR15, are critical for interactions.

**Figure 7:**
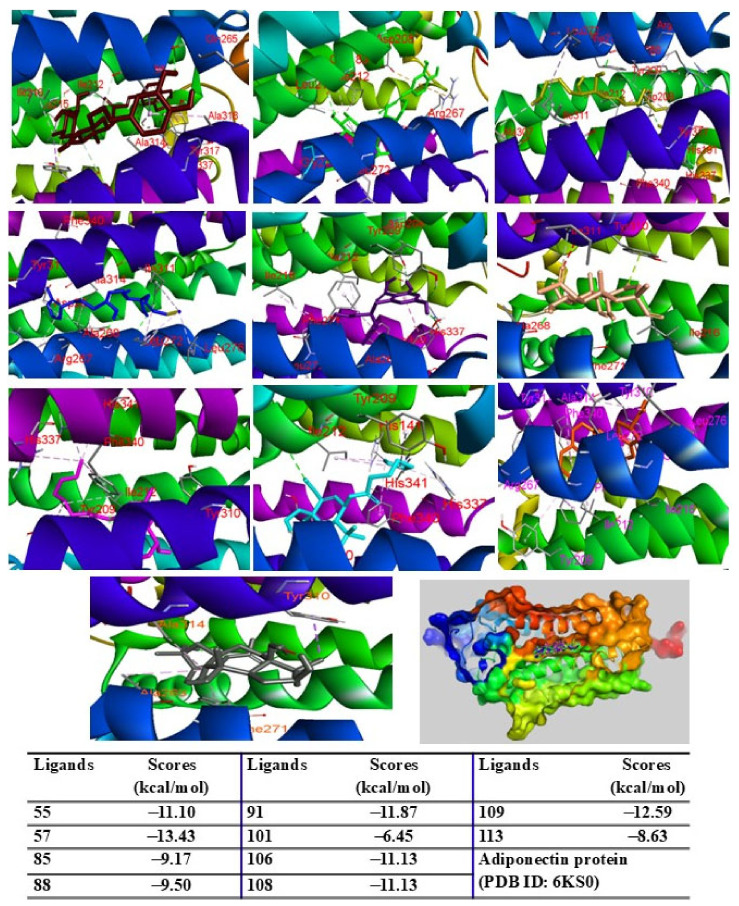
Interactions of compounds 55 (Raspberry), 57 (Green), 85 (Limon), 88 (Blue), 91 (Purple blue), 101 (Wheat), 106 (Magenta), 108 (Cyan), 109 (Orange), and 113 (Gray) with amino acid when they were docked into adiponectin protein (PDB ID: 6KS0).

**Figure 8:**
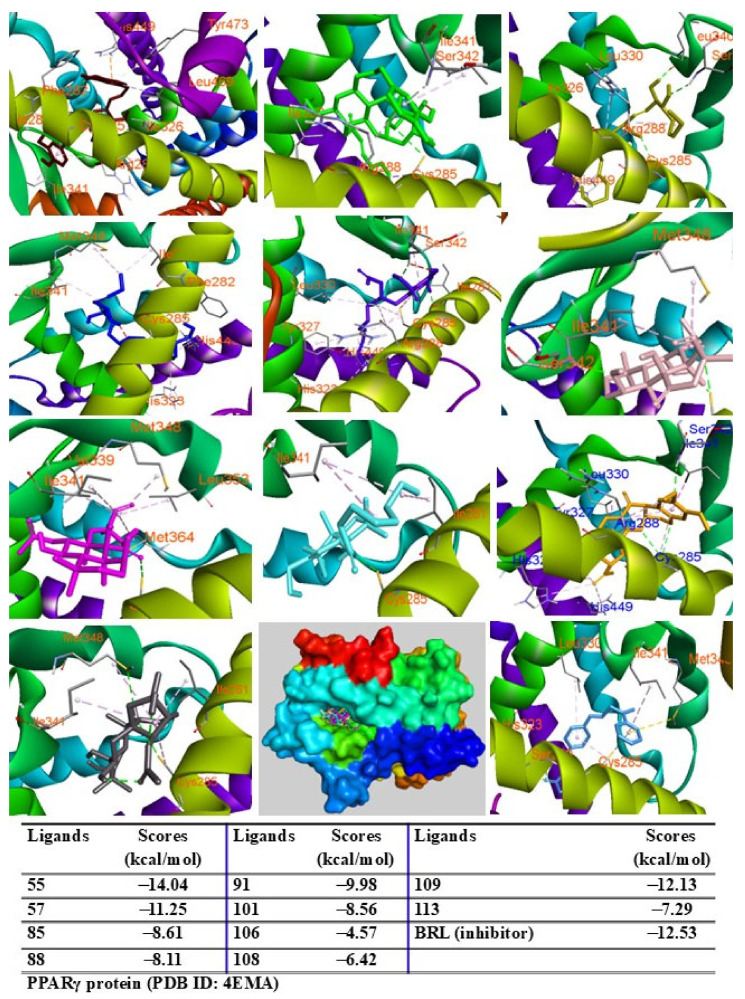
Interactions of compounds 55 (Raspberry), 57 (Green), 85 (Limon), 88 (Blue), 91 (Purple blue), 101 (Wheat), 106 (Magenta), 108 (Cyan), 109 (Orange), and 113 (Gray) with amino acid when they were docked into PPARγ protein (PDB ID: 4EMA).

When docked into the adiponectin protein, all major peaks were positioned within the same region of the ligand–protein binding pocket. Peak 57 displayed the lowest binding energy (G = −13.43 kcal/mol), followed by peaks 109, 91, 106, 108, and 55, with scores ranging from −12.59 to −11.10 kcal/mol. Peaks 85, 88, 113, and 101 showed binding energies between −9.50 kcal/mol and −6.45 kcal/mol (Figure 7). The major peaks also showed strong binding affinities for the PPARγ protein. Peaks 55, 57, and 109 demonstrated significant docking scores (G = −14.04, −11.25, and −12.13 kcal/mol, respectively), outperforming the redocked native ligand BRL (G = −12.53 kcal/mol). Peaks 85, 88, 91, 101, 108, and 113 exhibited binding affinities with scores ranging from −9.98 to −6.42 kcal/mol (Figure 8). When docked into iNOS protein, all major peaks occupied the binding pose within the ligand–protein complexes, confirming their potential as ligands for these targets.

**Figure 9:**
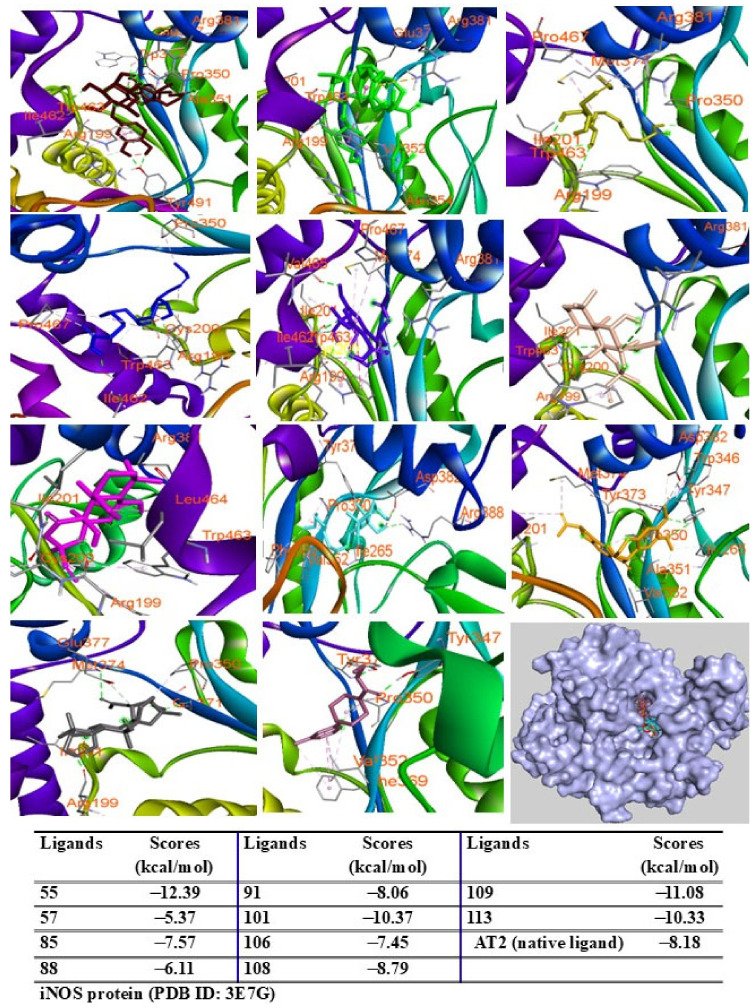
Interactions of compounds 55 (Raspberry), 57 (Green), 85 (Limon), 88 (Blue), 91 (Purple blue), 101 (Wheat), 106 (Magenta), 108 (Cyan), 109 (Orange), and 113 (Gray) with amino acids when they were docked into iNOS protein (PDB ID 5IKQ).

**Figure 10:**
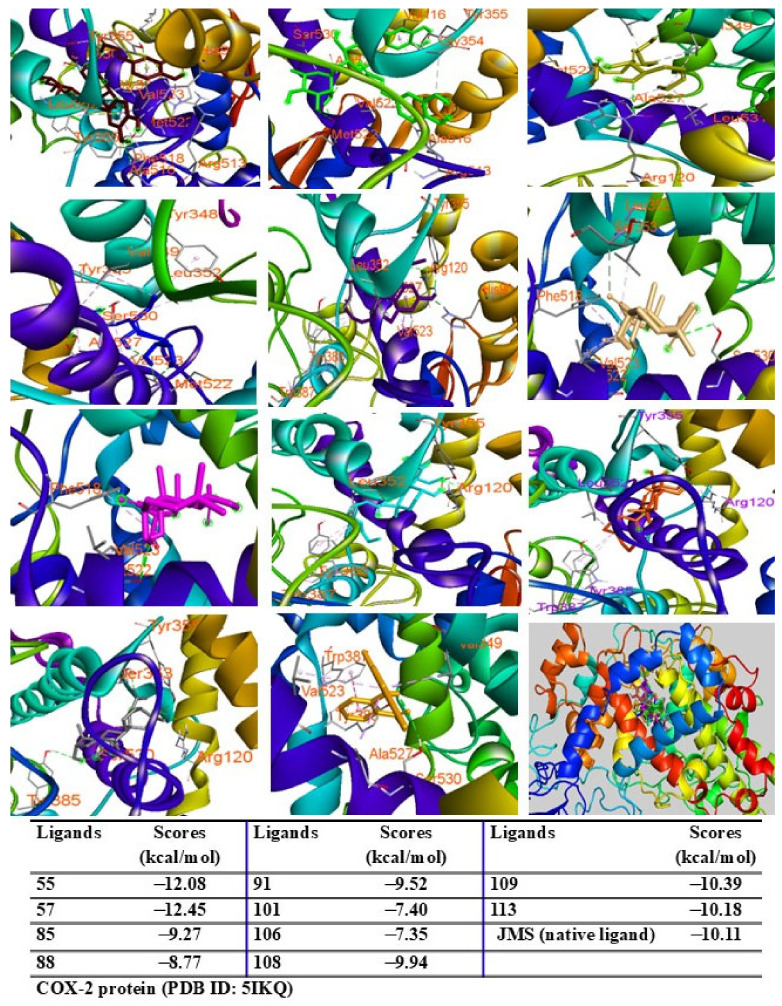
Interactions of compounds 55 (Raspberry), 57 (Green), 85 (Limon), 88 (Blue), 91 (Purple blue), 101 (Wheat), 106 (Magenta), 108 (Cyan), 109 (Orange), and 113 (Gray) with amino acids when they were docked into COX-2 protein (PDB ID 5IKQ).

Among the tested compounds, peaks 55, 91, 101, 108, 109, and 113 displayed significant binding scores (ΔG, from 12.39 to −8.06 kcal/mol) compared to the redocked native ligand (AT2, G = −8.18 kcal/mol) (Figure 9). These peaks also docked into the COX-2 protein, occupying the same binding pocket as the complexes. Peaks 55, 57, 109, and 113 demonstrated lower docking scores (G = −12.08, −12.45, −10.39, and −10.18 kcal/mol, respectively) than the redocked native ligand (JMS, G = −10.11 kcal/mol) (Figure 10).

**Figure 11:**
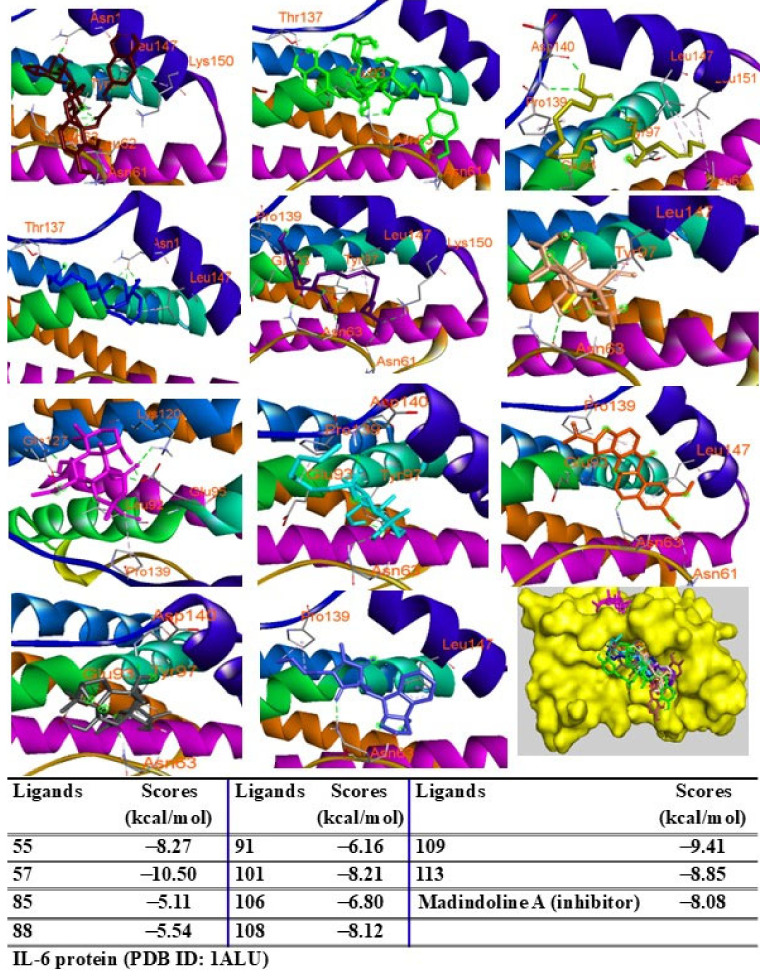
Interactions of compounds 55 (Raspberry), 57 (Green), 85 (Limon), 88 (Blue), 91 (Purple blue), 101 (Wheat), 106 (Magenta), 108 (Cyan), 109 (Orange), and 113 (Gray) with amino acids when they were docked into IL-6 protein (PDB ID: 1ALU).

**Figure 12:**
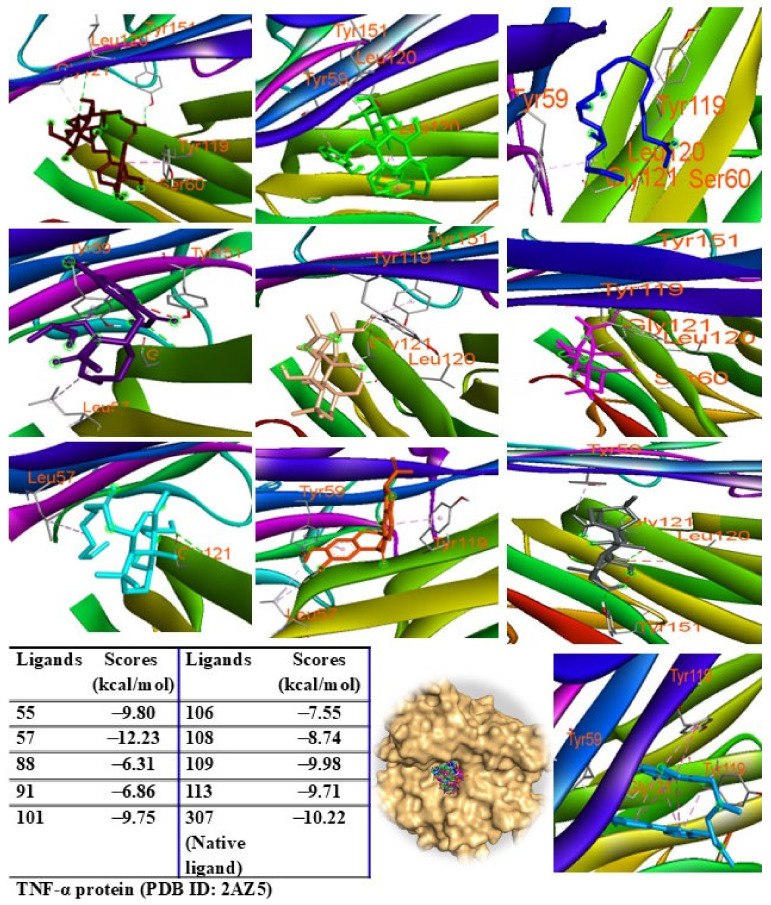
Interactions of compounds 55 (Raspberry), 57 (Green), 88 (Blue), 91 (Purple blue), 101 (Wheat), 106 (Magenta), 108 (Cyan), 109 (Orange), and 113 (Gray) with amino acids when they were docked into TNF-α protein (PDB ID: 2AZ5).

**Figure 13:**
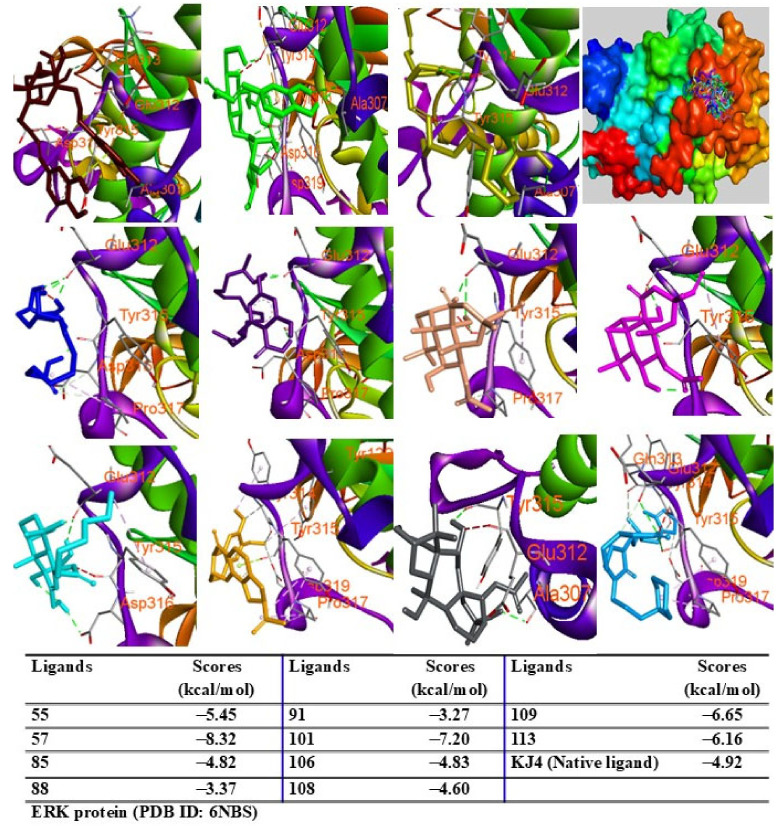
Interactions of compounds 55 (Raspberry), 57 (Green), 85 (Limon), 88 (Blue), 91 (Purple blue), 101 (Wheat), 106 (Magenta), 108 (Cyan), 109 (Orange), and 113 (Gray) with amino acids when they were docked into ERK protein (PDB ID: 6NBS).

Additionally, peaks 85, 91, and 108 showed significant scores (ΔG = −9.94 to −9.27 kcal/mol), outperforming the redocked ligand. When docked into IL-6 protein, most major peaks occupied the same binding pocket region as the IL-6 inhibitor madindoline A, except for peak 106. Peaks 55, 57, 101, 108, 109, and 113 displayed higher binding affinities (ΔG, ranging from −10.50 to −8.12 kcal/mol) compared to madindoline A (ΔG = −8.08 kcal/mol) (Figure 11). Similarly, these major peaks occupied the same binding poses within TNF-α protein complexes, with docking scores (ΔG) ranging from −12.23 to −6.31 kcal/mol, outperforming the redocked native ligand (307, G = −10.22 kcal/mol) (Figure 12). When docked into ERK protein, the major peaks exhibited significant binding scores (ΔG) ranging from −8.32 to −4.60 kcal/mol) exceeding the redocked native ligand (KJ4, G = −4.92 kcal/mol) (Figure 13). These results suggest that the major peaks exhibit strong binding affinity, potentially enhancing the bioactivities of the extract.

## 3. Discussion

Obesity, a global health crisis, has reached epidemic levels, characterized by excessive adipose tissue accumulation and significantly increased risks of chronic diseases, including type 2 diabetes, cardiovascular disease, and certain cancers [26,27]. The escalating prevalence of obesity in both developed and developing countries highlights the urgent need for innovative therapeutic strategies [28]. While lifestyle modifications, including dietary changes and physical activity, remain central to obesity management [29], achieving sustainable weight loss through these methods alone remains challenging for many individuals [30]. Pharmacological interventions provide additional support but often have limited efficacy and are associated with adverse side effects, underscoring the demand for safer and more effective alternatives [31,32].

Oxidative stress is intricately linked to chronic inflammation and metabolic dysregulation in obesity, positioning antioxidants as essential elements in therapeutic strategies. LSE demonstrated strong antioxidant activity in DPPH and ABTS assays, underscoring its potential to neutralize free radicals and mitigate oxidative damage. This activity is attributed to bioactive compounds such as chlorogenic acid, quercetin-3-*O*-galactoside, and ferulic acid, which are recognized for their potent antioxidant properties [33,34,35]. These effects may reduce oxidative stress in adipose tissue and macrophages, alleviating upstream triggers of chronic inflammation and enhancing LSE’s overall therapeutic potential.

In this study, we evaluated LSE as a dual-action therapeutic agent targeting both adipogenesis and inflammation. LSE significantly enhanced the differentiation and maturation of 3T3-L1 preadipocytes, particularly during the early and intermediate stages of differentiation (Figure 1 and Figure 2). This enhancement was accompanied by the upregulation of key adipogenic and lipogenic genes, including PPARγ, C/EBPα, and FABP4, indicating a regulatory role in adipocyte function. The observed increase in cyclin E expression suggests that LSE may also influence preadipocyte proliferation and mitotic clonal expansion, critical steps in effective adipogenesis. Notably, LSE did not affect CHOP expression, a negative regulator of adipogenesis, implying that it promotes differentiation through CHOP-independent mechanisms (Figure 3C,D). This contrasts with findings on Elsholtzia fruticosa (EF), which enhances adipogenesis by downregulating CHOP [36], suggesting that LSE and EF operate via distinct molecular pathways.

Additionally, this study revealed the potent anti-inflammatory properties of LSE in LPS-stimulated RAW 264.7 macrophages. Chronic, low-grade inflammation is a hallmark of obesity and its complications, including insulin resistance and cardiovascular disease [37]. LSE reduced the expression of pro-inflammatory mediators, such as TNF-α, IL-6, iNOS, and COX-2, and inhibited ERK phosphorylation, demonstrating its potential to attenuate macrophage-mediated inflammation and improve metabolic health (Figure 4).

To better understand the bioactive components of LSE, molecular network modeling and dereplication analyses identified eight major compound groups: glycosides, organic acids, terpenoids, flavonoids, phenylglycosides, phenolics, and fatty acids. Among these, the largest groups of flavonoids, phenolics, and organic acids demonstrated anti-inflammatory benefits by downregulating inflammatory cytokines and mediators [38,39,40]. Figure S2 highlights key compounds detected in the extract (Figure S2, Supplementary Materials), specifically geshoidin (4), (*S*)-malate (7), 6a,12a-didehydroamorphigenin (18), and 7-methyl-1-[(2*S*,3*R*,4*S*,5*S*,6*R*)-3,4,5-trihydroxy-6-(hydroxymethyl)oxan-2-yl]oxy-1,5,6,7a-tetrahydrocyclopenta[c]pyran-4a,5,7-triol (19), chlorogenic acid (23), 7-methyl-1-[(2*S*,3*R*,4*S*,5*S*,6*R*)-3,4,5-trihydroxy-6-(hydroxymethyl)oxan-2-yl]oxy-1,4a,5,6,7,7a-hexahydrocyclopenta[c]pyran-4-carboxylic acid (25), flavovilloside (41), kaempferol 3-*O*-neohesperidoside (56), acteoside (57), quercetin-3-*O*-galactoside (58), ferulic acid (59), (6*R*,9*S*)-3-oxo-α-ionol glucoside (61), quercetin-3-*O*-xyloside (64), (10*E*,15*Z*)-9,12,13-trihydroxyoctadeca-10,15-dienoic acid (85), (*Z*)-9,12,13-trihydroxyoctadec-15-enoic acid (88), 2-(2-acetyloxy-12-hydroxytridecyl)-4,6-dihydroxybenzoic acid (91), forskolin (101), 1-epi coleonol (106), [4,5-dihydroxy-3,4-bis(hydroxymethyl)-4a,8,8-trimethyl-5,6,7,8a-tetrahydro-1H-naphthalen-1-yl] octanoate (108), rotenone (109), and grayanotoxin I (113).

These compounds are likely contributors to the extract’s activity. Prior research confirmed chlorogenic acid, quercetin-3-*O*-galactoside, ferulic acid, quercetin-3-*O*-xyloside, and forskolin significantly reduced TNF-α, IL-6, and other cytokines in LPS-activated macrophages. Chlorogenic acid, quercetin-3-O-galactoside, ferulic acid, quercetin-3-*O*-xyloside, forskolin, and rotenone notably suppressed iNOS and COX-2 protein expression levels [41,42,43,44,45,46].

LSE also promoted adipocyte differentiation, with chlorogenic acid enhancing the differentiation of 3T3-L1 cells in animal models [47]. Forskolin predominantly upregulated PPARγ and C/EBPβ expression [35], while ferulic acid improved inflammation and insulin resistance via the NFκB/MAPK signaling pathways in adipocytes [48]. In silico analyses confirmed that major bioactive compounds interact with key amino acids in the binding pockets of target proteins, displaying varying binding affinities (Figures S4–S11, Supplementary Materials). These interactions likely underpin the biological activities of LSE, offering insights into its mechanisms of action.

Despite these encouraging findings, this study has limitations. First, the in vitro experiments cannot fully replicate complex in vivo physiological interactions. Future studies using animal models of obesity are necessary to validate the efficacy and safety of LSE in more physiologically relevant conditions. Second, the long-term effects and potential toxicity of LSE remain unexplored and warrant detailed investigation.

## 4. Materials and Methods

### 4.1. Methanol Extract of Lagopsis Supina (Stephan Ex Willd.) Ikonn.-Gal

The *L*. *supina* (Stephan ex Willd.) Ikonn.-Gal. extract (FBM188-007) used in this study was sourced from the International Biological Material Research Center at the Korea Research Institute of Bioscience and Biotechnology (KRIBB, Daejeon, Republic of Korea). The plant, collected in June 2013 from the Bornuur district, Tuv aimag province, Mongolia, is preserved as a voucher specimen (KRIB 0049867) in KRIBB’s herbarium. For extraction, 42 g of dried, shade-powdered whole plant and radix materials were immersed in 1 L of 99.9% methanol. The mixture underwent 30 cycles of ultrasonic extraction (SDN-900H, SD-ULTRASONIC CO., LTD., Seoul, Republic of Korea) at room temperature, with each cycle comprising 15 min of ultrasonication (40 kHz, 1500 W) and 120 min of standing. The extract was filtered (No. 100, HYUNDAI MICRO CO., LTD., Seoul, Republic of Korea) and concentrated under reduced pressure, yielding 3.16 g of LSE.

### 4.2. DPPH and ABTS Assays

The antioxidant capacity of LSE was assessed through two complementary assays. The DPPH (2,2-diphenyl-1-picrylhydrazyl) free radical scavenging activity was measured at 517 nm using an iMarkTM Microplate Reader (Bio-Rad Laboratories Inc., Hercules, CA, USA), where decreased absorbance indicated increased scavenging activity. ABTS radical scavenging activity was evaluated by measuring absorbance at 743 nm, with reduced absorbance reflecting a lower radical concentration. Ascorbic acid (AA) served as positive control in both assays.

### 4.3. Cell Culture and Adipocyte Differentiation

Mouse 3T3-L1 preadipocytes and RAW 264.7 macrophages were procured from the American Type Culture Collection (ATCC, Manassas, VA, USA). 3T3-L1 cells were cultured in Dulbecco’s Modified Eagle Medium (DMEM; Welgene, Gyeongsan-si, Republic of Korea) supplemented with 10% bovine calf serum, while RAW 264.7 macrophages were maintained in DMEM containing 10% fetal bovine serum (FBS; Welgene Inc.), 100 U/mL penicillin, and 100 μg/mL streptomycin. All cells were incubated at 37 °C in a humidified atmosphere with 5% CO_2_. For adipocyte differentiation, 3T3-L1 preadipocytes were seeded at 5 × 10^4^ cells per well in 12-well plates [49]. Upon reaching confluence (Day 0, D0), the medium was replaced with a differentiation medium comprising DMEM, 10% FBS, and a 0.17 × MDI hormonal cocktail. This cocktail contained 0.17 mM 3-isobutyl-1-methylxanthine (M; Sigma Aldrich, St. Louis, MO, USA), 0.33 μM dexamethasone (D; Sigma Aldrich), and 0.33 μg/mL insulin (I; Sigma Aldrich). To induce terminal differentiation, cells were cultured from Day 2 (D2) onward in DMEM with 10% FBS and 0.2 μg/mL insulin, with the medium refreshed every 48 h until Day 6 (D6). Differentiation into mature adipocytes was verified microscopically by observing rounded cell morphology and intracellular lipid droplets.

### 4.4. Cell Viability Assay

Cell viability was evaluated using a WST-8-based cell counting kit (BIOMAX, Seoul, Republic of Korea) per the manufacturer’s protocol. This colorimetric assay measures metabolic activity by quantifying the conversion of tetrazolium salt (WST-8) into a water-soluble formazan product by mitochondrial dehydrogenases. In brief, 3T3-L1 and RAW 264.7 cells were seeded into 96-well plates at densities of 1.0 × 10^4^ and 1.0 × 10^5^ cells/well, respectively, and cultured for 24 h. Cells were then treated with varying concentrations of LSE for 24 h. After treatment, WST-8 reagent was added, and plates were incubated for 1–2 h. Absorbance at 450 nm was measured using an iMarkTM Microplate Reader (Bio-Rad Laboratories Inc.), and cell viability was expressed as a percentage relative to untreated controls, set at 100% viability.

### 4.5. NO Production Assay in RAW 264.7 Cells

RAW 264.7 macrophages, seeded at 1.0 × 10^5^ cells/well in a 96-well plate, were allowed to adhere for 24 h. Upon reaching confluence, cells were treated with different LSE concentrations in DMEM supplemented with 10% FBS without phenol red. Dimethyl sulfoxide (DMSO) was used as a vehicle control, and N-acetylcysteine (NAC, 10 mM) served as a positive control due to its anti-inflammatory effects. After a 2 h pretreatment, lipopolysaccharide (LPS, 0.1 ng/mL) was added to stimulate NO production, followed by 24 h of incubation at 37 °C. Supernatants were collected, centrifuged at 2000 rpm for 10 min at 4 °C, and transferred to new 96-well plates. Equal volumes of supernatant and Griess reagent were mixed (1:1 ratio) following the manufacturer’s protocol. Absorbance at 560 nm was measured using an iMark™ Microplate Reader (Bio-Rad Laboratories Inc.). Nitrite concentration, an indirect marker of NO production, was determined from a standard curve prepared with a commercial NO standard.

### 4.6. Oil Red O Staining for Lipid Droplets

Following differentiation, 3T3-L1 adipocytes cultured in 12-well plates were washed twice with Dulbecco’s phosphate-buffered saline to remove residual media. The cells were fixed with 3.7% formalin at room temperature for 10 min to maintain cellular morphology. After fixation, they were washed twice with 100% propylene glycol on a shaker at 30 rpm, with each wash lasting 30 min to ensure dehydration. The cells were then stained with 1 mL of 0.7% Oil red O solution for 15 min. Oil red O, a lipophilic dye with a strong affinity for neutral lipids, stained intracellular lipid droplets bright red. Excess dye was removed by a brief wash with 85% propylene glycol for 1 min, followed by thorough rinsing with distilled water. Stained lipid droplets were observed under a microscope (Nexcope NIB410, Ningbo, China) at 200× magnification.

### 4.7. RNA Isolation and Quantitative Real-Time PCR (qRT-PCR) Analysis

Total RNA was extracted from cells using the RiboEx™ reagent (GeneAll Biotechnology, Seoul, Republic of Korea), and complementary DNA (cDNA) was synthesized from the isolated RNA with the ReverTra Ace™ qPCR RT kit (Toyobo, Osaka, Japan) following the manufacturer’s instructions. Gene expression levels were quantified by quantitative real-time PCR (qRT-PCR) using the CFX Connect Real-Time PCR System and SFCgreen PCR Master Mix (Biofact, Deajeon, Republic of Korea). Beta-actin and 36B4 were used as internal controls to normalize the expression data. Primer sequences for most target genes were obtained from previous studies [22,23], with the following exceptions: β-actin (F) 5′-GGC TGT ATT CCC CTC CAT CG-3′ and (R) 5′-CCA GTT GGT AAC AAT GCC ATG-3′; TNF-α (F) 5′-CAT CTT CTC AAA ATT CGA GT-3′ and (R) 5′-TGG GAG TAG ACA AGG TAC AA-3′; iNOS (F) 5′-CTC AGC CCA ACA ATA CAA GAT-3′ and (R) 5′-TGT GGT GAA GAG TGT CAT GCA-3′; COX-2 (F) 5′-GAA GTC TTT GGT CTG GTG CGT G-3′ and (R) 5′-GTC TGC TGG TTT GGA ATA GTT GC-3′; IL-6 (F) 5′-TAG TCC TTC CTA CCC CAA TTT CC-3′ and (R) 5′-TTG GTC CTT AGC CAC TCC TTC-3′.

### 4.8. Western Blot Analysis

Cell lysates were prepared using a mammalian protein extraction reagent containing protease and phosphatase inhibitors (GenDEPOT, Baker, TX, USA). After centrifugation at 13,000 rpm for 15 min at 4 °C, the supernatants were collected. Protein concentrations were determined using the BCA assay kit (Thermo Scientific, Rockford, IL, USA, Cat. No. 23227). Equal amounts of protein were resolved by SDS-PAGE and transferred to polyvinylidene difluoride (PVDF) membranes. The membranes were blocked with 5% BSA in TBST and incubated overnight at 4 °C with primary antibodies targeting the following proteins: PPARγ (Cell Signaling Technology, Danvers, MA, USA, Cat. No. 2443), adiponectin (Thermo Scientific, Cat. No. MA1-054), CHOP (Cell Signaling Technology, Cat. No. 2895), HSP90 (Santa Cruz Biotechnology, Dallas, TX, USA, Cat. No. SC-13119), iNOS (Cell Signaling Technology, Cat. No. 2443), COX-2 (Thermo Scientific, Cat. No. MA1-054), p44/42 MAPK (ERK1/2) (Cell Signaling Technology, Cat. No. 9102), phospho-p44/43 MAPK (ERK1/2) (Cell Signaling Technology, Cat. No. 4370), and β-actin (Santa Cruz Biotechnology, Cat. No. 8432). After washing, membranes were incubated with horseradish peroxidase (HRP)-conjugated secondary antibodies (Bio-Rad Laboratories Inc.) for 1 h at room temperature. Protein bands were visualized using enhanced chemiluminescence (ECL, Bio-Rad Laboratories Inc.) and imaged with the iBright CL1500 imaging system (Thermo Scientific).

### 4.9. Transfection and Luciferase and β-galactosidase Assay

Transfection was carried out using LipofectaminTM 3000 (Thermo Fisher Scientific) according to the manufacturer’s instructions. For the luciferase reporter assay, cells were cotransfected with the mammalian expression vector pGL3-DR-1-basic as the reporter construct, pCMV-β-galactosidase for normalization of transfection efficiency, and expression vectors for PPARγ and RXRα where applicable. Luciferase and β-galactosidase activities were measured as previously described [50]. Luminescence from luciferase activity was detected using a MicroLumat Plus LB96V luminometer (Berthold Technologies, Bad Wildbad, Germany), and β-galactosidase activity was measured with an Infinite^®^ 200 PRO (Tecan, Männedorf, Switzerland). Luciferase activity was reported in relative light units and normalized to β-galactosidase activity to control variations in transfection efficiency.

### 4.10. UPLC-ESI-Orbitrap MS/MS

The LC-MS/MS analysis was performed using a Vanquish UHPLC system coupled with an Orbitrap Exploris 120 mass spectrometer (Thermo Fisher Scientific, Sunnyvale, CA, USA) [51]. Chromatographic separation was conducted on a Waters Acquity UPLC HSS T3 column (4.6 × 100 mm, 1.8 μm, Waters, Milford, MA, USA) at 40 °C, with a flow rate of 0.2 mL/min and an injection volume of 4 μL. A gradient solvent system was used for mobile phase elution: phase A (water containing 0.1% formic acid) and phase B (ACN, 0.1% formic acid) were applied in the following sequence: 5%–15% (B) for 0–4 min, 15%–35% (B) for 4–12 min, 35%–45% (B) for 12–17 min, 45%–100% (B) for 17–23 min (held for 3 min), and 100%–5% (B) for 1 min before returning to 5% (B) for re-equilibration. Prior to analysis, sample solutions were filtered through a 0.22 μm PTFE membrane (Agilent Technologies, Santa Clara, CA, USA).

The MS data were acquired using electrospray ionization (ESI) in negative ion mode with the following settings: spray voltage, 3.5 kV; sheath gas (N_2_) > 50%; auxiliary gas (N_2_) > 15%; heater temperature, 350 °C; capillary temperature, 320 °C. In full MS scanning mode, the mass range was m/z 100–1500, with a resolution of 120,000, and in-source collision-induced dissociation (in-source CID) was set to 0 eV. For MS/MS scanning, data-dependent MS^2^ (dd-MS^2^) was performed with a resolution of 30,000, and high-energy collision-induced dissociation was used in stepped mode (15 eV, 30 eV, 60 eV).

### 4.11. Molecular Networking

The molecular network of the LSE was constructed using the Global Natural Products Social Molecular Networking (GNPS) online platform (https://gnps.ucsd.edu, accessed on 15 June 2024) [1]. Briefly, raw data from the UHPLC−ESI-MS/MS analysis were converted into mzXML format using the MSConvert tool from ProteoWizard (https://proteowizard.sourceforge.io/, accessed on 12 June 2024). Data processing was carried out using MZmine 3.9.0, following a previously described method [24]. Molecular networks were generated through the GNPS workflow (https://ccms-ucsd.github.io/GNPSDocumentation/, accessed on 15 June 2024, job ID: 4475ce44b79a414ea9cd2a57c737e14b), and the resulting network data were downloaded from GNPS and visualized using Cytoscape 3.9.1 (https://www.cytoscape.org/, accessed on 18 June 2024). The composition of compounds was relatively quantitated by accessing the peak intensity of those detected from the chromatograms.

### 4.12. LC-MS Annotation

Component identification from the extracts and fractions was performed using open-source software, including GNPS web tools and MS-Dial, alongside public mass spectrometry databases (GNPS, HMDB, LipidMaps, KNApSAcK, and the American Mass Bank). Compound annotations were made by comparing precursor and fragmentation ion spectra with those in public MS/MS databases for spectral matching.

### 4.13. Molecular Docking Assay

The crystal structures of target proteins, including antioxidative (1HCK), adiponectin (PDB ID: 6KS0), PPARγ (PDB ID: 4EMA), IL-6 (PDB ID: 1ALU), iNOS (PDB ID: 3E7G), COX-2 (PDB ID: 5IKQ), TNF-α (PDB ID: 2AZ5), and ERK (PDB ID: 6NBS), were retrieved from the RCSB Protein Data Bank (https://www.rcsb.org; accessed 18 July 2024, 23 July 2024, and 25 July 2024). Protein structures were processed using MGL Tools 1.5.6, which involved removing water and heteroatoms, adding polar hydrogens and Kollman charges, and saving the prepared structures in pdbqt format. Compound structures were downloaded from PubChem (https://pubchem.ncbi.nlm.nih.gov; accessed 10 July 2024, 15 July 2024, 22 July 2024, and 30 July 2024) in SDF format. Missing structures were manually created using the Avogadro package, and all structures were converted to pdbqt format using the Open Babel program version 2.4.1. Ligands were prepared in MGL Tools 1.5.6 by adding Gasteiger charges. Grid box parameters were defined using PyMol (version 3.1.1), and the Lamarckian genetic algorithm in AutoDock 4.2.6 was applied to find the optimal ligand conformation. The resulting protein–ligand complexes were analyzed and visualized using Discovery Studio Visualizer 2021 and PyMol [52].

### 4.14. Statistical Analysis

Data are expressed as the mean ± standard error of the mean (SEM) to represent the average value and variability within each treatment group. Sample size (*n*) for each experiment is provided for transparency. Statistical analyses were performed using either Microsoft Excel or GraphPad Prism software version 10.4.1. Comparisons between two groups were made using an unpaired, two-tailed Student’s *t*-test. For experiments involving multiple treatment groups, one-way analysis of variance was followed by Tukey’s post hoc test to identify significant differences between specific groups. Statistical significance was defined as a *p*-value < 0.05.

## 5. Conclusions

This study underscores the therapeutic potential of *Lagopsis supina* (LSE) extract for obesity and related complications. LSE exhibited robust antioxidant activity, promoted adipocyte differentiation via the upregulation of transcription factors like PPARγ, and enhanced lipogenesis through genes such as FABP4 and SCD1. It also demonstrated notable anti-inflammatory effects in LPS-stimulated macrophages by downregulating pro-inflammatory mediators and inhibiting ERK phosphorylation, addressing metabolic dysregulation and inflammation. Chemical analyses revealed bioactive components, including flavonoids and phenolic acids, that were likely responsible for these effects. Molecular docking provided mechanistic insights, highlighting strong interactions between these compounds and key proteins in antioxidant, adipogenic, and inflammatory pathways. However, the in vitro nature of this study emphasizes the need for in vivo validation to confirm efficacy and safety. Further research on LSE’s long-term effects, bioavailability, and synergy with existing therapies is crucial to fully harness its therapeutic potential. In conclusion, LSE offers a novel approach to obesity treatment by addressing metabolic and inflammatory factors, positioning it as a promising candidate for clinical applications.

## Figures and Tables

**Table 1:** Identification of metabolites from LSE.

No.	Compound	RT (min)	Formula	Adduct	*m*/*z* (Da)	Error (mDa)	Class
1	Unknown	1.080	-	[M-H]^−^	386.9382	-	-
2	Unknown	1.090	-	[M-H]^−^	272.9587	-	-
3	Unknown	1.090	-	[M-H]^−^	158.9782	-	-
4	Geshoidin	1.273	C_18_H_18_O_9_	[M-H]^−^	377.0853	2.496	Glycosides
5	Quinic acid ^†^	1.304	C_7_H_12_O_6_	[M-H]^−^	191.0559	0.172	Organic acids
6	Unknown	1.310	-	[M-H]^−^	404.1040	-	-
7	(*S*)-malate	1.342	C_4_H_6_O_5_	[M-H]^−^	133.0140	0.168	Organic acids
8	N-Fructosyl pyroglutamate	1.349	C_11_H_17_NO_8_	[M-H]^−^	290.0879	0.782	Organic acids
9	Sucrose	1.379	C_12_H_22_O_11_	[M-H]^−^	341.1083	0.651	Glycosides
10	5-Oxo-D-proline	1.902	C_5_H_7_NO_3_	[M-H]^−^	128.0352	0.103	Organic acids
11	Citrate	1.902	C_6_H_8_O_7_	[M-H]^−^	191.0194	0.258	Organic acids
12	Succinate	2.187	C_4_H_6_O_4_	[M-H]^−^	117.0192	0.061	Organic acids
13	5,9-dihydroxy-5,7,7-trimethyl-4,5a,6,8,8a,9-hexahydro-1H-azuleno[5,6-c]furan-3-one	2.221	C_15_H_22_O_4_	[M-H]^−^	265.0928	1.156	Sesquiterpenes
14	1-*O*-Caffeoylglucose	2.221	C_15_H_18_O_9_	[M-H]^−^	341.0873	0.295	Glycosides
15	(1*S*,6*S*,13*S*)-13-Hydroxy-16,17-dimethoxy-6-prop-1-en-2-yl-2,7,20-trioxapentacyclo[11.8.0.03,11.04,8.014,19]henicosa-3(11),4(8),9,14,16,18-hexaen-12-one	2.327	C_23_H_22_O_7_	[M-H]^−^	409.1349	4.917	Isoflavonoids
16	3-(4-Hydroxyphenyl)lactate	3.155	C_9_H_10_O_4_	[M-H]^−^	181.0506	0.032	Organic acids
17	Unknown	3.155	-	[M-H]^−^	361.1137	-	-
18	6a,12a-Didehydroamorphigenin	3.155	C_23_H_20_O_7_	[M-H]^−^	407.1183	4.768	Isoflavonoids
19	7-methyl-1-[(2*S*,3*R*,4*S*,5*S*,6*R*)-3,4,5-trihydroxy-6-(hydroxymethyl)oxan-2-yl]oxy-1,5,6,7a-tetrahydrocyclopenta[c]pyran-4a,5,7-triol	4.053	C_15_H_24_O_10_	[M-H]^−^	363.1290	0.588	Glycosides
20	Benzoic acid ^†^	6.132	C_13_H_16_O_8_	[M-H]^−^	137.0243	0.206	Organic acids
21	Neochlorogenic acid ^†^	6.154	C_16_H_18_O_9_	[M-H]^−^	353.0873	1.699	Organic acids
22	[(2*R*,3*S*,4*S*,5*R*,6*S*)-6-(3,4-dihydroxybenzoyl)oxy-3,4,5-trihydroxyoxan-2-yl]methyl 3,4-dihydroxybenzoate	6.189	C_20_H_20_O_12_	[M-H]^−^	451.2175	4.395	Phenylglycosides
23	Chlorogenic acid ^†^	6.652	C_167_H_18_O_9_	[M-H]^−^	353.0872	0.824	Phenolics
24	2-[(4-Hydroxy-3,5-dimethoxyphenyl)methoxy]-6-(hydroxymethyl)oxane-3,4,5-triol	6.936	C_15_H_22_O_9_	[M-H]^−^	345.1186	0.459	Glycosides
25	7-methyl-1-[(2*S*,3*R*,4*S*,5*S*,6*R*)-3,4,5-trihydroxy-6-(hydroxymethyl)oxan-2-yl]oxy-1,4a,5,6,7,7a-hexahydrocyclopenta[c]pyran-4-carboxylic acid	6.936	C_16_H_24_O_9_	[M-H]^−^	405.1397	0.480	Terpenoids
26	9,13-Dihydroxy-4-megastigmen-3-one 9-glucoside	7.152	C_19_H_32_O_8_	[M+HCOO]^−^	433.2071	1.882	Terpenoids
27	4-(*β*-D-Glucopyranosyloxy)benzyl 2,3-dihydroxy-3-methylbutanoate	7.220	C_18_H_26_O_10_	[M+HCOO]^−^	447.1503	0.436	Organic acids
28	7-*Epi*-12-hydroxyjasmonic acid glucoside	7.371	C_18_H_28_O_9_	[M-H]^−^	387.1655	0.551	Glycosides
29	Epigallocatechin ^†^	7.395	C_15_H_14_O_7_	[M-H]^−^	305.0699	3.288	Flavanols
30	5-hydroxy-2,2,6,6-tetramethyl-4-[2-methyl-1-[2,4,6-trihydroxy-3-(2-methylpropanoyl)phenyl]propyl]cyclohex-4-ene-1,3-dione	7.406	C_24_H_32_O_7_	[M-H]^−^	431.1915	4.226	Fatty acids
31	Methyl 2,4,10-triacetyloxy-5,9-dihydroxy-1,4a-dimethyl-7-propan-2-yl-2,3,4,9,10,10a-hexahydrophenanthrene-1-carboxylate	7.406	C_27_H_36_O_10_	[M-H]^−^	519.2437	0.771	Sesquiterpenes
32	Aesculetin	7.486	C_9_H_6_O_4_	[M-H]^−^	177.0190	0.244	Coumarins
33	Oregonoyl A	7.509	C_33_H_36_O_12_	[M-H]^−^	623.2188	4.846	Phenylglycosides
34	Chrysophanol	7.531	C_15_H_10_O_4_	[M-H]^−^	253.0713	2.260	Quinones
35	Phenylacetic acid	7.676	C_8_H_8_O_2_	[M-H]^−^	135.0451	0.048	Organic acids
36	3-(4-Hydroxyphenyl)pyruvic acid	7.676	C_9_H_8_O_4_	[M-H]^−^	179.0347	0.249	Organic acids
37	Unknown	7.709	-	[M-H]^−^	601.2348	-	-
38	Bonanniol A	7.742	C_25_H_28_O_6_	[M-H]^−^	423.1866	4.618	Dihydroflavonols
39	(2E)-4-Hydroxy-3,4-dimethyl-2-penten-1-yl 6-O-[(2S,3R,4R)-3,4-dihydroxy-4-(hydroxymethyl)tetrahydro-2-furanyl]-β-D-glucopyranoside	7.742	C_18_H_32_O_11_	[M+HCOO]^−^	469.1921	0.527	Glycosides
40	1H-3,9a-Methanocyclopent[c]oxocin-4-carboxylic acid, 3,4,5,6,6a,9-hexahydro-4-hydroxy-7-(1-hydroxy-1-methylethyl)-	7.753	C_15_H_22_O_5_	[M-H]^−^	281.1389	0.477	Organic acids
41	Flavovilloside	7.786	C_33_H_40_O_20_	[M-H]^−^	755.2026	1.337	Flavonoid glycosides
42	Strobide B	7.831	C_11_H_10_O_7_	[M-H]^−^	253.0351	0.233	Phenolics
43	Manghaslin	8.213	C_33_H_40_O_20_	[M-H]^−^	755.2034	0.549	Flavonoid glycosides
44	2,3-dihydroxy-4-methoxy-4′-ethoxybenzophenone	8.214	C_16_H_16_O_5_	[M-H]^−^	287.1500	3.949	Fatty acids
45	3-*O*-Feruloylquinic acid ^†^	8.378	C_33_H_40_O_20_	[M-H]^−^	367.1028	0.183	Organic acids
46	Caffeic acid ^†^	8.410	C_9_H_8_O_4_	[M-H_2_O-H]^−^	179.0347	0.170	Organic acids
47	Unknown	8.410	-	[M-H]^−^	591.0980	-	-
48	Vanillylmandelic acid	8.419	C_9_H_10_O_5_	[M-H]^−^	197.0454	0.100	Organic acids
49	[(2*S*,3*S*)-2-[(*E*,3*R*,4*S*)-3,4-dihydroxypent-1-enyl]-6-oxo-2,3-dihydropyran-3-yl] (*E*)-2-methylbut-2-enoate	8.419	C_15_H_20_O_6_	[M-H]^−^	295.1184	0.242	Monoterpenoids
50	(-)-12-hydroxyjasmonic acid	8.584	C_12_H_18_O_4_	[M-H]^−^	225.1133	0.414	Fatty acids
51	Coumaroyl quinic acid ^†^	8.752	C_16_H_18_O_8_	[M-H]^−^	337.0927	0.702	Organic acids
52	3-[4,5-dihydroxy-3-[(2*R*,3*R*,4*R*,5*R*,6*S*)-3,4,5-trihydroxy-6-methyloxan-2-yl]oxy-6-[[(2*R*,3*R*,4*R*,5*R*,6*S*)-3,4,5-trihydroxy-6-methyloxan-2-yl]oxymethyl]oxan-2-yl]oxy-5,7-dihydroxy-2-(4-hydroxyphenyl)chromen-4-one	8.808	C_33_H_40_O_19_	[M-H]^−^	739.2083	0.671	Flavonoid glycosides
53	Quercetin 3-*O*-*α*-rhamnopyranosyl-(1-2)-*β*-galactopyranoside	8.899	C_27_H_30_O_16_	[M-H]^−^	609.1454	0.860	Flavonoid glycosides
54	Rutin ^†^	9.405	C_27_H_30_O_16_	[M-H]^−^	609.1454	0.321	Flavonoid glycosides
55	[(2*R*,3*R*,4*R*,5*R*,6*R*)-2-[[(2*R*,3*R*,4*R*)-3,4-dihydroxy-4-(hydroxymethyl)oxolan-2-yl]oxymethyl]-4-[(2*S*,3*R*,4*R*,5*R*,6*S*)-4,5-dihydroxy-6-methyl-3-[(2*S*,3*R*,4*S*,5*S*)-3,4,5-trihydroxyoxan-2-yl]oxyoxan-2-yl]oxy-6-[2-(3,4-dihydroxyphenyl)ethoxy]-5-hydroxyoxan-3-yl] (*E*)-3-(3,4-dihydroxyphenyl)prop-2-enoate	9.415	C_34_H_44_O_19_	[M-H]^−^	755.2395	1.025	Phenylglycosides
56	Kaempferol 3-*O*-neohesperidoside ^†^	9.596	C_30_H_26_O_13_	[M-H]^−^	593.1505	0.488	Flavonoid glycosides
57	Acteoside	9.705	C_29_H_36_O_15_	[M-H]^−^	623.1972	1.668	Phenylglycosides
58	Quercetin-3-*O*-galactoside	9.896	C_21_H_20_O_12_	[M-H]^−^	463.0875	1.265	Flavonoid glycosides
59	Ferulic acid ^†^	10.343	C_10_H_10_O_4_	[M-H]^−^	193.0504	0.224	Phenolics
60	Quercetin-3-*O*-pentosyl(1-2)acetylpentoside	10.386	C_27_H_28_O_16_	[M-H]^−^	607.1293	1.106	Flavonoid glycosides
61	(6*R*,9*S*)-3-o*xo*-α-ionol glucoside	10.517	C_19_H_30_O_7_	[M+HCOO]^−^	415.1970	0.371	Terpenoids
62	[6-[2-(3,4-dihydroxyphenyl)ethoxy]-2-(hydroxymethyl)-4-(3,4,5-trihydroxy-6-methyloxan-2-yl)oxy-5-(3,4,5-trihydroxyoxan-2-yl)oxyoxan-3-yl] (*E*)-3-(4-hydroxy-3-methoxyphenyl)prop-2-enoate	10.529	C_35_H_46_O_19_	[M-H]^−^	769.2544	1.693	Phenylglycosides
63	Unknown	10.716	-	[M-H]^−^	629.2650	-	-
64	Quercetin-3-*O*-xyloside	10.774	C_20_H_18_O_11_	[M-H]^−^	433.0769	1.099	Flavonoid glycosides
65	3-[(2*S*,3*R*,4*R*,5*S*)-3,4-dihydroxy-5-(hydroxymethyl)oxolan-2-yl]oxy-2-(3,4-dihydroxyphenyl)-5,7-dihydroxychromen-4-one	10.791	C_20_H_18_O_11_	[M-H]^−^	433.07714	0.501	Flavonoid glycosides
66	Kaempferol-3-*O*-glucoside ^†^	10.825	C_21_H_20_O_11_	[M-H]^−^	447.09260	1.587	Flavonoid glycosides
67	[(2*R*,3*S*,4*S*,5*R*,6*S*)-6-(3,4-dihydroxybenzoyl)oxy-3,4,5-trihydroxyoxan-2-yl]methyl 3,4-dihydroxybenzoate	11.142	C_20_H_20_O_12_	[M-H]^−^	451.2175	4.396	Phenylglycosides
68	Salidroside	11.185	C_14_H_20_O_7_	[M-H]^−^	299.1138	0.188	Glycosides
69	6″-*O*-(3-Hydroxy-3-methylglutaroyl)astragalin	11.426	C_27_H_28_O_15_	[M-H]^−^	591.1347	0.491	Glycosides
70	Azelaic acid ^†^	11.545	C_9_H_16_O_4_	[M-H]^−^	187.0974	0.026	Organic acids
71	6-[(6,8-dihydroxy-7-methoxy-3-methyl-1-oxo-3,4-dihydroisochromen-4-yl)oxy]-4,8-dihydroxy-7-methoxy-3-methyl-3,4-dihydroisochromen-1-one	11.741	C_22_H_22_O_11_	[M-H]^−^	461.1085	0.392	Quinones
72	Kaempferol-3-*O*-arabinoside ^†^	11.842	C_20_H_18_O_10_	[M-H]^−^	417.0822	0.794	Flavonoid glycosides
73	*β*-D-Glucopyranoside, 2-(3,4-dimethoxyphenyl)ethyl 3-*O*-(6-deoxy-alpha-L-mannopyranosyl)-4-*O*-[(2*E*)-3-(3,4-dihydroxyphenyl)-1-oxo-2-propen-1-yl]-6-*O*-[(2*S*,3*R*,4*R*)-tetrahydro-3,4-dihydroxy-4-(hydroxymethyl)-2-furanyl]-	11.886	C_36_H_48_O_19_	[M-H]^−^	783.2706	1.131	Phenylglycosides
74	(5*E*)-3,4,9-trihydroxy-2-propyl-2,3,4,7,8,9-hexahydrooxecin-10-one	11.986	C_12_H_20_O_5_	[M-H]^−^	243.1237	0.047	Polyketides
75	Tricin 5-glucoside	12.020	C_23_H_24_O_12_	[M-H]^−^	491.1197	0.233	Flavonoid glycosides
76	Corylifol A	12.032	C_25_H_26_O_4_	[M-H]^−^	389.1808	4.979	Flavonoids
77	2-Hydroxyanthraquinone	12.050	C_14_H_8_O_3_	[M-H]^−^	223.0371	2.994	Phenols
78	Phyllanthin ^†^	12.153	C_24_H_34_O_6_	[M-H]^−^	417.2485	3.298	Phenylglycosides
79	[5-hydroxy-6-[2-(4-hydroxy-3-methoxyphenyl)ethoxy]-2-(hydroxymethyl)-4-(3,4,5-trihydroxy-6-methyloxan-2-yl)oxyoxan-3-yl] (*E*)-3-(4-hydroxy-3-methoxyphenyl)prop-2-enoate	12.377	C_31_H_40_O_15_	[M-H]^−^	651.2297	0.266	Phenylglycosides
80	[6-[2-(3,4-dihydroxyphenyl)-8-hydroxy-4-oxochromen-7-yl]oxy-3,4,5-trihydroxyoxan-2-yl]methyl (*E*)-3-(4-hydroxyphenyl)prop-2-enoate	13.741	C_30_H_26_O_13_	[M-H]^−^	593.1293	0.451	Flavonoid glycosides
81	Tribuloside	14.111	C_30_H_26_O_13_	[M-H]^−^	593.1293	0.732	Flavonoid glycosides
82	Unknown	14.289	-	[M-H]^−^	507.2697	-	-
83	Apigenin-7-neohesperidoside	14.559	C_27_H_30_O_14_	[M-H]^−^	577.1638	3.592	Flavonoid glycosides
84	Procyanidin B2	14.943	C_30_H_26_O_12_	[M-H]^−^	577.1346	0.451	Proanthocyanidin
85	(10*E*,15*Z*)-9,12,13-trihydroxyoctadeca-10,15-dienoic acid	15.402	C_18_H_32_O_5_	[M-H]^−^	327.2173	0.880	Fatty acids
86	[(2*R*,3*S*,4*S*,5*R*,6*S*)-6-[2,3-dihydroxy-4-[(*E*)-3-(4-hydroxyphenyl)prop-2-enoyl]phenoxy]-3,4,5-trihydroxyoxan-2-yl]methyl (*E*)-3-(4-hydroxyphenyl)prop-2-enoate	15.452	C_30_H_28_O_12_	[M-H]^−^	579.1497	0.571	Chalcones
87	1-(3,5-Dihydroxyphenyl)-12-hydroxytridecan-2-one	15.531	C_19_H_30_O_4_	[M+HCOO]^−^	367.2118	0.806	Fatty acids
88	(*Z*)-9,12,13-trihydroxyoctadec-15-enoic acid	16.462	C_18_H_34_O_5_	[M-H]^−^	329.2329	0.984	Fatty acids
89	Caperatic acid	16.512	C_21_H_38_O_7_	[M-H]^−^	803.5136	2.373	Organic acids
90	Unknown	16.824	-	[M-H]^−^	473.2751	-	-
91	2-(2-Acetyloxy-12-hydroxytridecyl)-4,6-dihydroxybenzoic acid	17.308	C_22_H_34_O_7_	[M-H]^−^	409.2223	1.304	Organic acids
92	Unknown	17.308	-	[M-H]^−^	819.4519	-	-
93	2-(2-Hydroxybut-3-en-2-yl)-3a,6,6,9a-tetramethyl-2,4,5,5a,7,8,9,9b-octahydro-1H-benzo[e][1]benzofuran-4,5-diol	17.523	C_20_H_34_O_4_	[M-H]^−^	337.2383	0.076	Organic acids
94	[4,5-Dihydroxy-3,4-bis(hydroxymethyl)-4a,8,8-trimethyl-5,6,7,8a-tetrahydro-1H-naphthalen-1-yl] hexanoate	17.523	C_21_H_36_O_6_	[M-H]^−^	383.2437	0.180	Fatty acids
95	Unknown	18.063	-	[M-H]^−^	441.2488	-	-
96	Aceroside VIII	18.131	C_30_H_42_O_12_	[M-H]^−^	593.2626	2.264	Glycosides
97	Unknown	18.295	-	[M-H]^−^	473.2752	-	-
98	9,10-Epoxy-13-*oxo*-11-octadecenoic acid	18.343	C_18_H_30_O_4_	[M-H]^−^	309.2070	1.042	Fatty acids
99	Unknown	18.372	-	[M-H]^−^	491.2750	-	-
100	2-(3-(8-Hydroxyoctyl)phenoxy)-2-methylpropanoic acid	19.466	C_18_H_28_O_4_	[M-H]^−^	307.1910	0.409	Fatty acids
101	Forskolin	19.687	C_22_H_34_O_7_	[M-H]^−^	409.2223	0.763	Diterpenes
102	3″,4″-Di-*O*-*p*-coumaroylafzelin	19.870	C_39_H_32_O_14_	[M-H]^−^	723.1699	1.943	Flavonoids
103	Unknown	20.081	-	[M-H]^−^	489.2597	-	-
104	3-[(2*S*,3*R*,4*S*,5*S*,6*R*)-3-[(2*S*,3*R*,4*R*)-3,4-dihydroxy-4-(hydroxymethyl)oxolan-2-yl]oxy-4,5-dihydroxy-6-(hydroxymethyl)oxan-2-yl]oxy-5-hydroxy-2-(4-hydroxyphenyl)-7-[(2*S*,3*R*,4*R*,5*R*,6*S*)-3,4,5-trihydroxy-6-methyloxan-2-yl]oxychromen-4-one	20.148	C_32_H_38_O_19_	[M-H]^−^	725.1878	5.683	Flavonoid glycosides
105	Biochanin A	20.437	C_16_H_12_O_5_	[M-H]^−^	283.0607	0.407	Isoflavone
106	1-*Epi* coleonol	20.475	C_22_H_34_O_7_	[M-H]^−^	409.2224	0.793	Terpenoids
107	Amorfrutin	20.581	C_18_H_26_O_4_	[M-H]^−^	305.1754	0.378	Terpenoids
108	[4,5-dihydroxy-3,4-bis(hydroxymethyl)-4a,8,8-trimethyl-5,6,7,8a-tetrahydro-1H-naphthalen-1-yl] octanoate	21.023	C_23_H_40_O_6_	[M-H]^−^	457.2798	0.867	Fatty acids
109	Rotenone	21.292	C_23_H_22_O_6_	[M-H]^−^	393.2271	0.143	Isoflavone
110	Nandrolone	22.280	C_18_H_26_O_2_	[M-H_2_O-H]^−^	275.2015	1.519	Steroids
111	15,16-Epoxy-9,12-octadecadienoic acid	22.290	C_18_H_30_O_3_	[M-H]^−^	293.2120	0.271	Fatty acids
112	2-Hydroxy-4,5′,8a′-trimethyl-1′-oxo-4-vinyloctahydro-1′H-spiro[cyclopentane-1,2′-naphthalene]-5′-carboxylic acid	22.588	C_20_H_30_O_4_	[M-H]^−^	333.2071	0.005	Organic acids
113	Grayanotoxin I	22.830	C_22_H_36_O_7_	[M-H_2_O-H]^−^	393.2276	0.176	Diterpenes
114	9-Hydroxy-10*E*,12*Z*-octadecadienoic acid	22.962	C_18_H_32_O_3_	[M-H]^−^	295.2272	0.636	Fatty acids

^†^ Reference standard. Other metabolites were identified by analyzing and comparing precursor and fragmentation ions to those obtained from public MS/MS databases for spectral matching.

## Data Availability

Data are contained within the article and Supplementary Materials.

## Supplementary Materials

The following supporting information can be downloaded at https://www.mdpi.com/article/10.3390/ph18020150/s1.
