# Supplementary material for: Chemical Composition and Biological Activities of Lagopsis supina Extract: Antioxidant, Adipogenic, and Ani-Inflammatory Effects
Source: Pharmaceuticals (Basel). 2025 Jan 23;18(2):150. doi: 10.3390/ph18020150 (PMC11860115; doi:10.3390/ph18020150)
Supplement: Supplementary file 1 [file pharmaceuticals-18-00150-s001.zip › pharmaceuticals-3426218-supplementary.pdf]

Supplementary materials

Chemical Composition and Biological Activities of *Lagopsis supina* Extract: Antioxidant, Adipogenic, and Anti-Inflammatory Effects

Juhyun Choi [1,†], Duc Dat Le [2,†], Nayoung Noh [1], Jiseok Lee [1], Shrestha Deumaya [3], Thientam Dinh [2], Vinhquang Truong [2], Badamtsetseg Bazarragchaa [4], Soo-Yong Kim [5], Sung-Suk Suh [1,3], Mina Lee [2,6,*], and Jong Bae Seo[1,3,*]

[1] Department of Biomedicine, Health & Life Convergence Sciences, BK21 Four, Biomedical and Healthcare Research Institute, Mokpo National University, Muan 58554, Jeonnam, Republic of Korea.

[2] College of Pharmacy and Research Institute of Life and Pharmaceutical Sciences, Sunchon National University, 255 Jungangno, Suncheon 57922, Jeonnam, Korea.

[3] Department of Biosciences, Mokpo National University, Muan 58554, Jeonnam, Republic of Korea

[4] Natural History Museum of Mongolia, Ulaanbaatar 15141, Mongolia.

[5] International Biological Material Research Center, Korea Research Institute of Bioscience and Biotechnology, Daejeon 34141, Republic of Korea.

[6] Department of Natural Cosmetics Science and Natural Cosmetics Research Institute, Sunchon National Univeristy, 255 Jungangno, Suncheon 57922, Jeonnam, Republic of Korea.

[†]These authors contributed equally to this work

*****Correspondences:

Jong Bae Seo, jbseo@mokpo.ac.kr; Tel.: +82-61-450-2348; Fax: +82-61-450-2349

Mina Lee, minalee@scnu.ac.kr; Tel.: +82-61-750-3764; Fax: +82-61-750-3708

Table of Contents

Figure S1. Effects of LSE on viability and differentiation conditions on 3T3-L1 cells....1
Figure S2. 2D and 3D chromatograms of LSE……………….……….…………….…..……2
Figure S3. Composition of peak areas of compounds of LSE…………...………………3
Figure S4. Interactions of compounds **55** (Raspberry), **57** (Green), **85** (Limon), **88** (Blue), **91** (Purple blue), **101** (Wheat), **106** (Magenta), **108** (Cyan), **109** (Orange), and **113** (Gray), with amino acid when they were docked into adiponectin protein (**PDB ID: 6KS0**). ………………………………………………………………………………………4
Figure S5. Interactions of compounds **55** (Raspberry), **57** (Green), **85** (Limon), **88** (Blue), **91** (Purple blue), **101** (Wheat), **106** (Magenta), **108** (Cyan), **109** (Orange), and **113** (Gray), with amino acid when they were docked into adiponectin protein (**PDB ID: 6KS0**). ………………………………………………………………………………………5
Figure S6. Interactions of compounds **55** (Raspberry), **57** (Green), **85** (Limon), **88** (Blue), **91** (Purple blue), **101** (Wheat), **106** (Magenta), **108** (Cyan), **109** (Orange), and **113** (Gray), with amino acid when they were docked into PPARγ protein (**PDB ID: 4EMA**). ……………………………………………………………………………………….6
Figure S7. Interactions of compounds **55** (Raspberry), **57** (Green), **85** (Limon), **88** (Blue), **91** (Purple blue), **101** (Wheat), **106** (Magenta), **108** (Cyan), **109** (Orange), and **113** (Gray), with amino acid when they were docked into iNOS protein (**PDB ID 3E7G**). ………………………………………………………………………………………7
Figure S8. Interactions of compounds **55** (Raspberry), **57** (Green), **85** (Limon), **88** (Blue), **91** (Purple blue), **101** (Wheat), **106** (Magenta), **108** (Cyan), **109** (Orange), and **113** (Gray), with amino acid when they were docked into COX-2 protein (**PDB ID 5IKQ**). ………………………………………………………………………………………8
Figure S9. Interactions of compounds **55** (Raspberry), **57** (Green), **85** (Limon), **88** (Blue), **91** (Purple blue), **101** (Wheat), **106** (Magenta), **108** (Cyan), **109** (Orange), and **113** (Gray), with amino acid when they were docked into IL-6 protein (**PDB ID: 1ALU**). .... ………………………………………………………….......................................9
Figure S10. Interactions of compounds **55** (Raspberry), **57** (Green), **88** (Blue), **91** (Purple blue), **101** (Wheat), **106** (Magenta), **108** (Cyan), **109** (Orange), and **113** (Gray), with amino acid when they were docked into TNF-α protein (**PDB ID: 2AZ5**). ……………………………………..……………………………………………10
Figure S11. Interactions of compounds **55** (Raspberry), **57** (Green), **85** (Limon), **88** (Blue), **91** (Purple blue), **101** (Wheat), **106** (Magenta), **108** (Cyan), **109** (Orange), and **113** (Gray), with amino acid when they were docked into ERK protein (**PDB ID: 6NBS**). ………………………………………………………………………….…..11

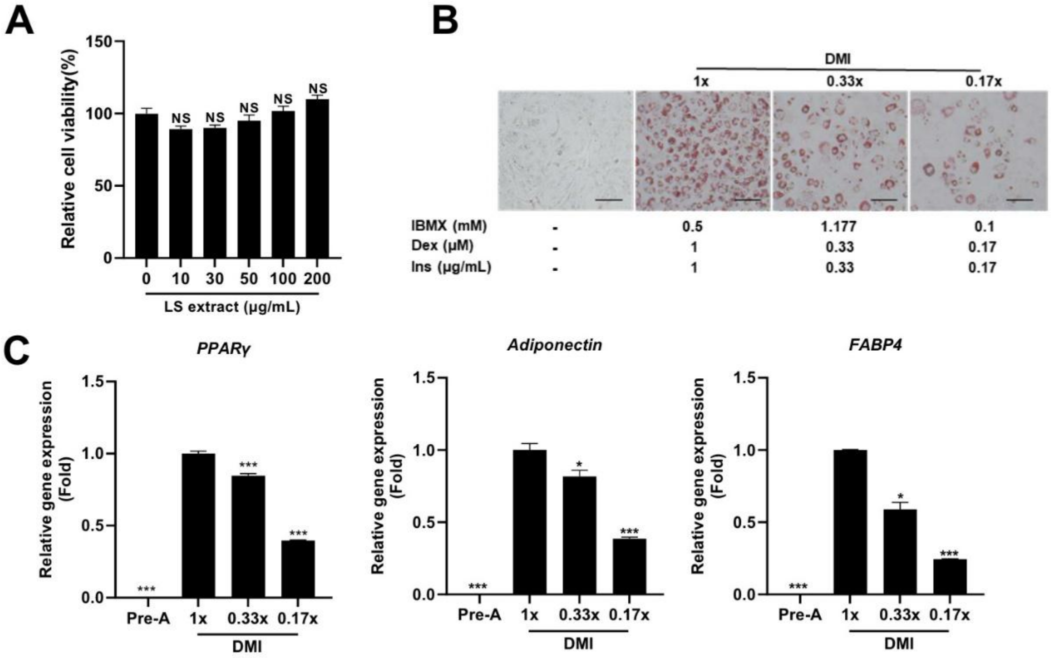

Figure S1. Effects of LSE on viability and differentiation conditions on 3T3-L1 cells. (**A**) The effect of LSE on 3T3-L1 cell viability was evaluated by treating the cells with varying concentrations of LSE for 24 hours, followed by a WST-8 assay. (**B**) 3T3-L1 cells were induced to differentiate into adipocytes for 6 days using media containing different concentrations of IBMX, dexamethasone, and insulin (DMI) at 0.17×, 0.33×, or 1× strength. Lipid accumulation was visualized using Oil red O staining. (**C**) qRT-PCR analysis was performed to measure mRNA expression levels of adipogenic genes, including *PPARγ*, *adiponectin*, and *FABP4*. The scale bar represents 100 μm (magnification, ×200). Abbreviations: IBMX, 3-isobutyl-1-methylxanthine; Dex., dexamethasone; Ins., insulin. Data are presented as mean ± SEM from two independent experiments (n = 4). Statistical significance is denoted as ***$p < 0.001$ versus adipocyte (1×) group; "ns" indicates non-significant differences.

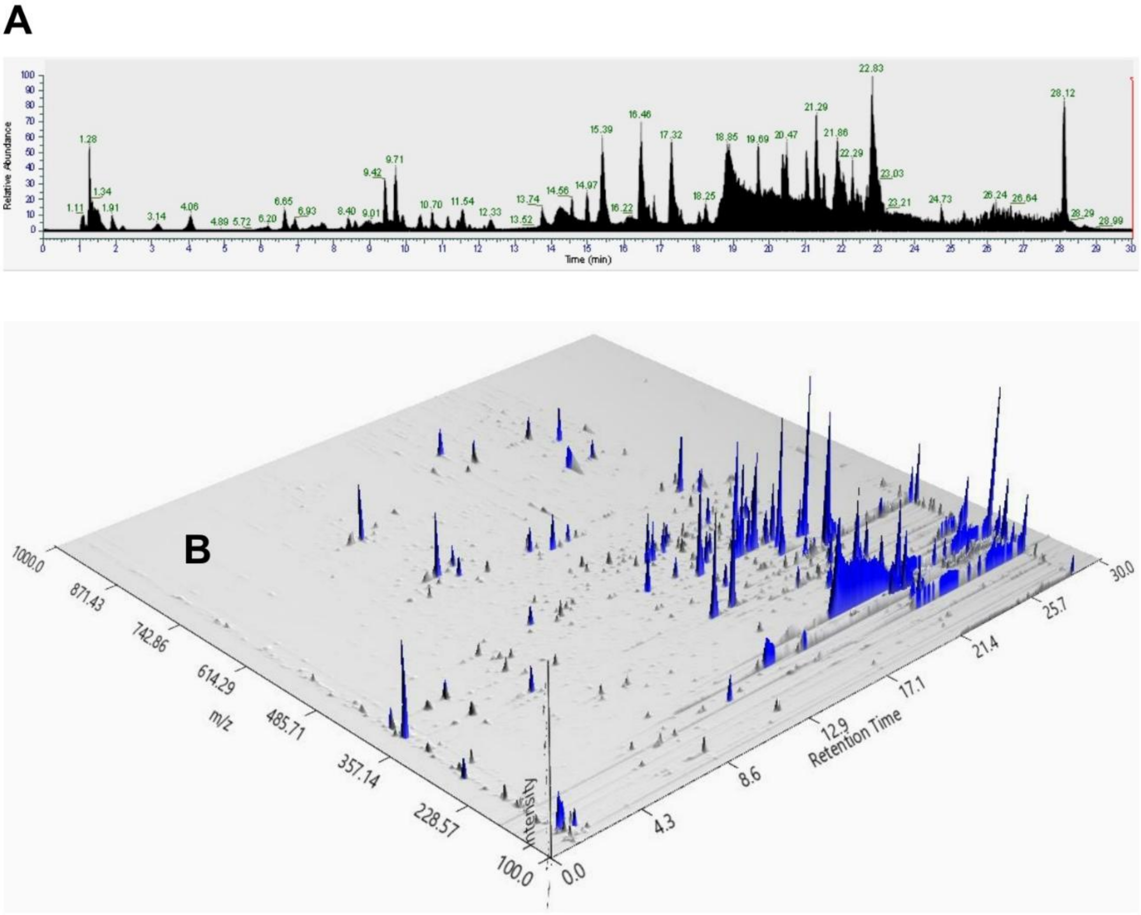

Figure S2. 2D (A) and 3D (B) chromatograms of compounds of LSE.

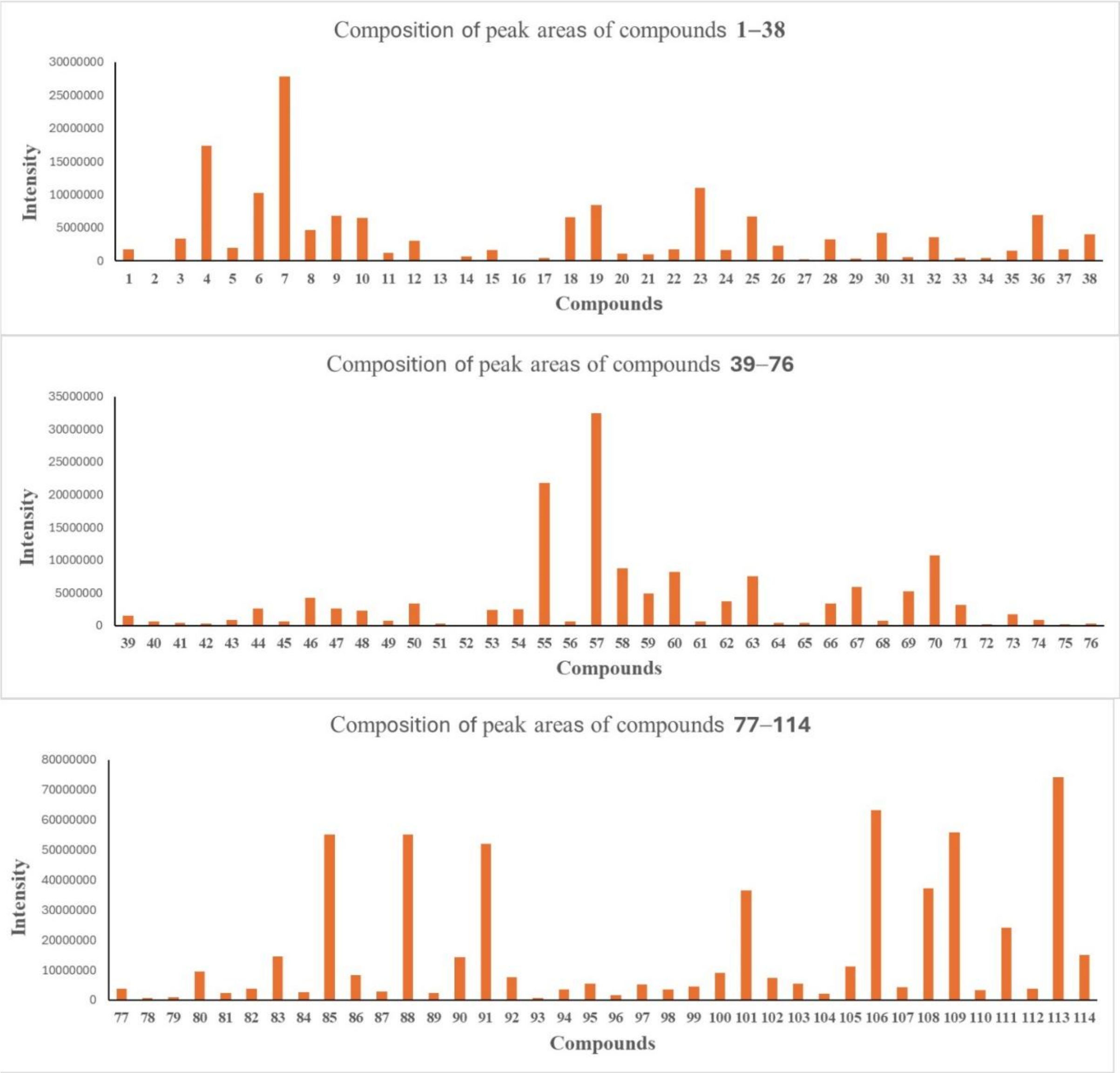

Figure S3. Composition of peak areas of compounds of LSE.

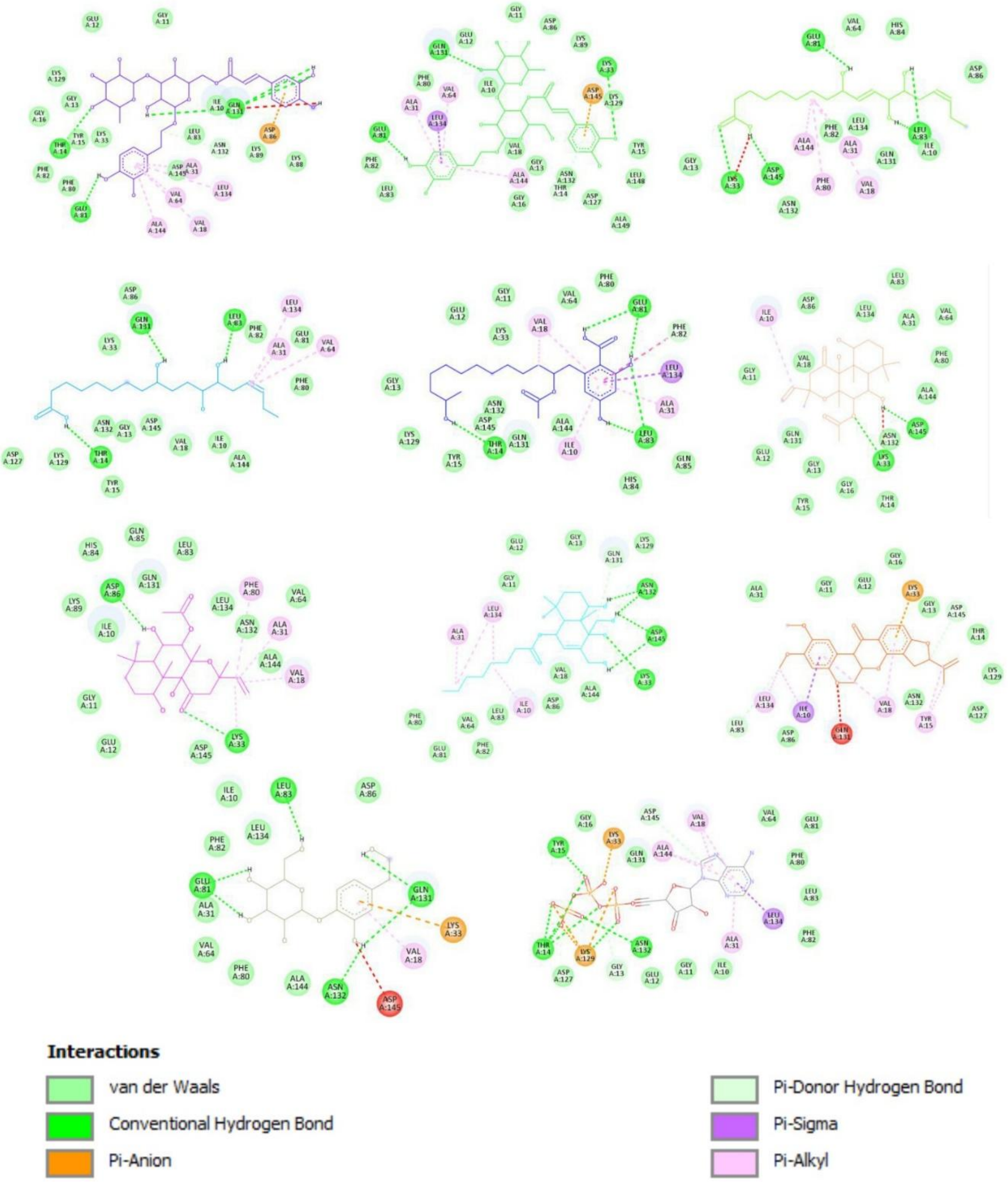

Figure S4. Interactions of compounds **55** (Raspberry), **57** (Green), **85** (Limon), **88** (Blue), **91** (Purple blue), **101** (Wheat), **106** (Magenta), **108** (Cyan), **109** (Orange), and **113** (Gray), with amino acid when they were docked into protein (**PDB ID: 1HCK**).

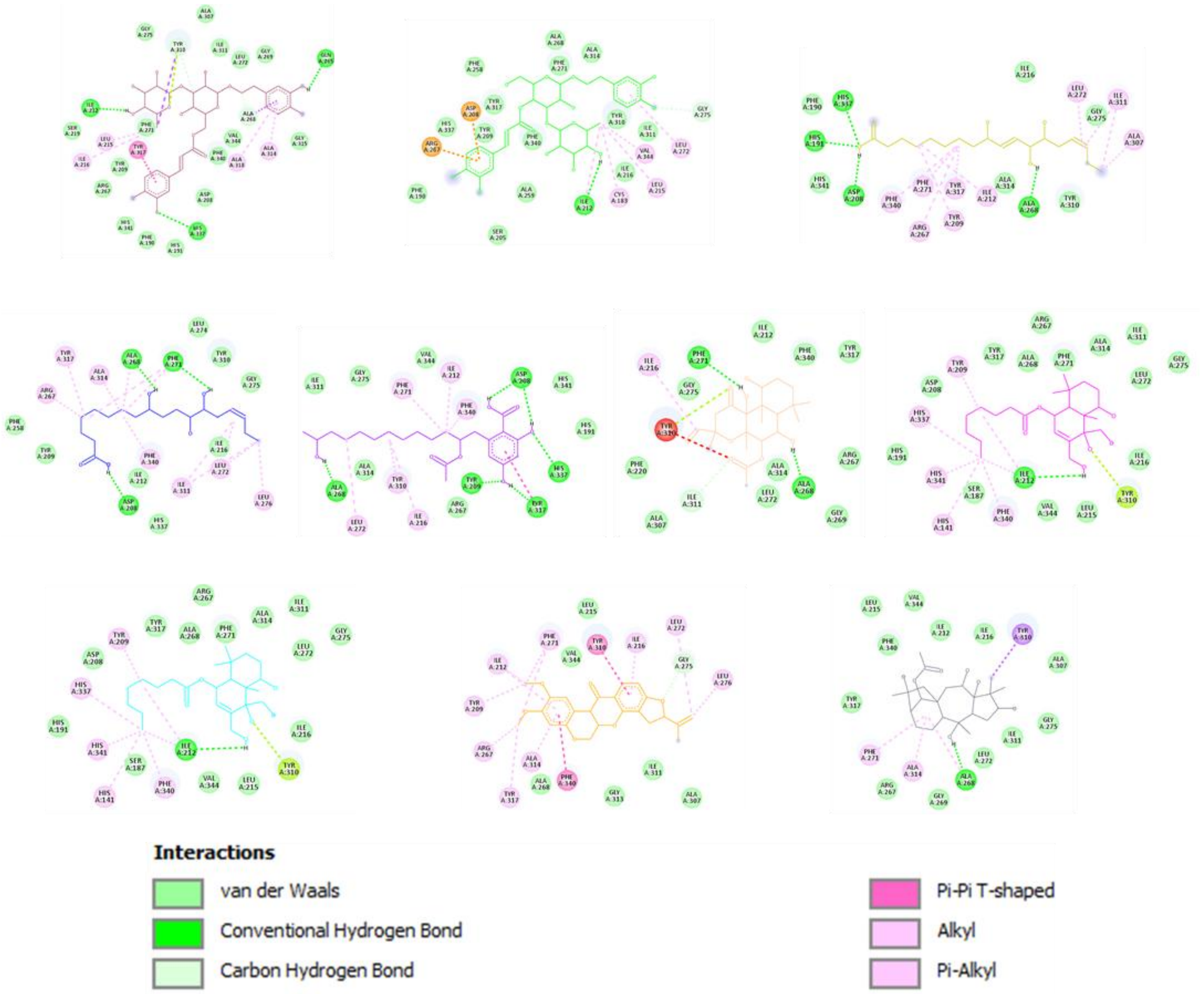

Figure S5. Interactions of compounds **55** (Raspberry), **57** (Green), **85** (Limon), **88** (Blue), **91** (Purple blue), **101** (Wheat), **106** (Magenta), **108** (Cyan), **109** (Orange), and **113** (Gray), with amino acid when they were docked into adiponectin protein (**PDB ID: 6KS0**).

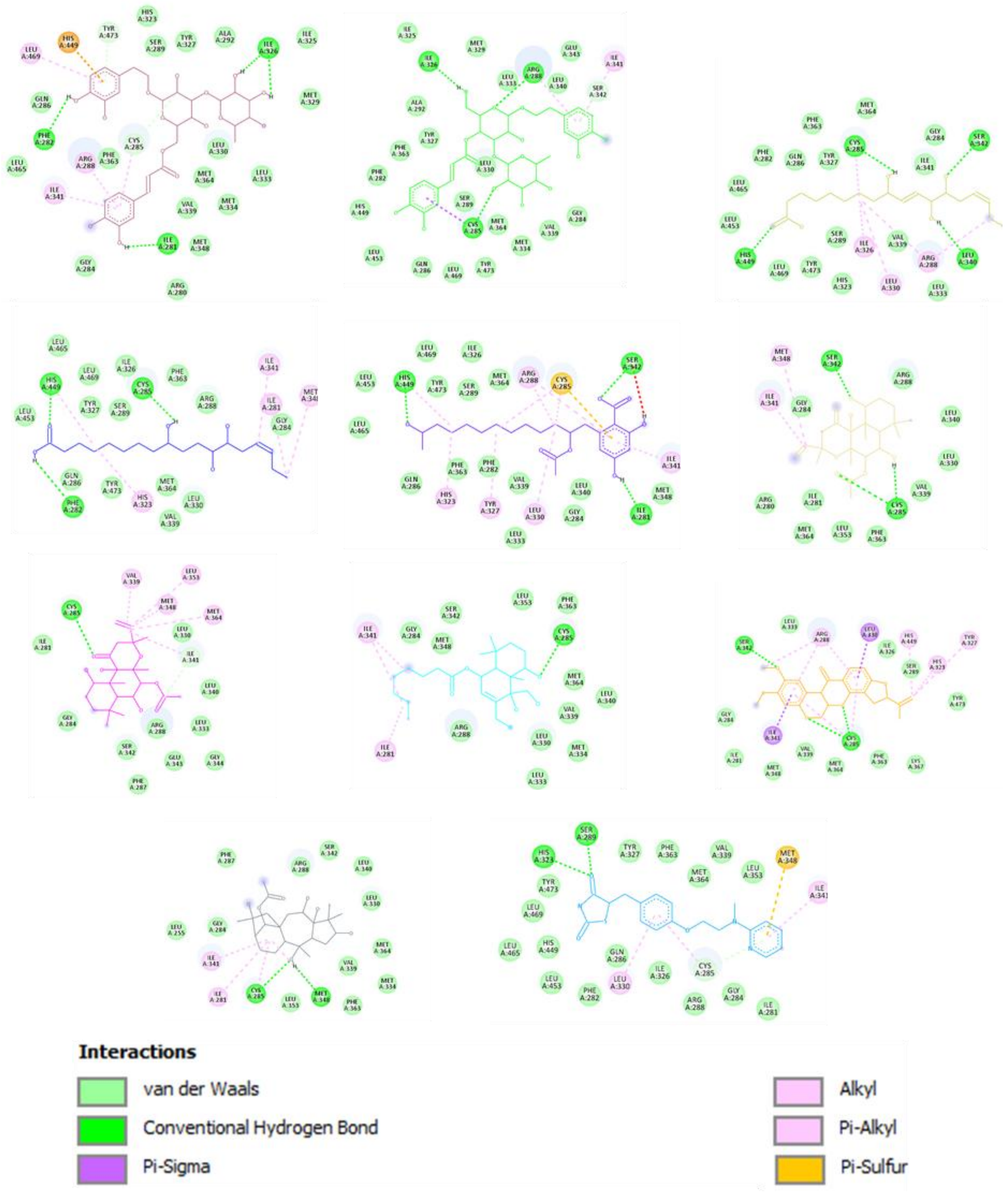

Figure S6. Interactions of compounds **55** (Raspberry), **57** (Green), **85** (Limon), **88** (Blue), **91** (Purple blue), **101** (Wheat), **106** (Magenta), **108** (Cyan), **109** (Orange), and **113** (Gray), with amino acid when they were docked into PPARγ protein (**PDB ID: 4EMA**).

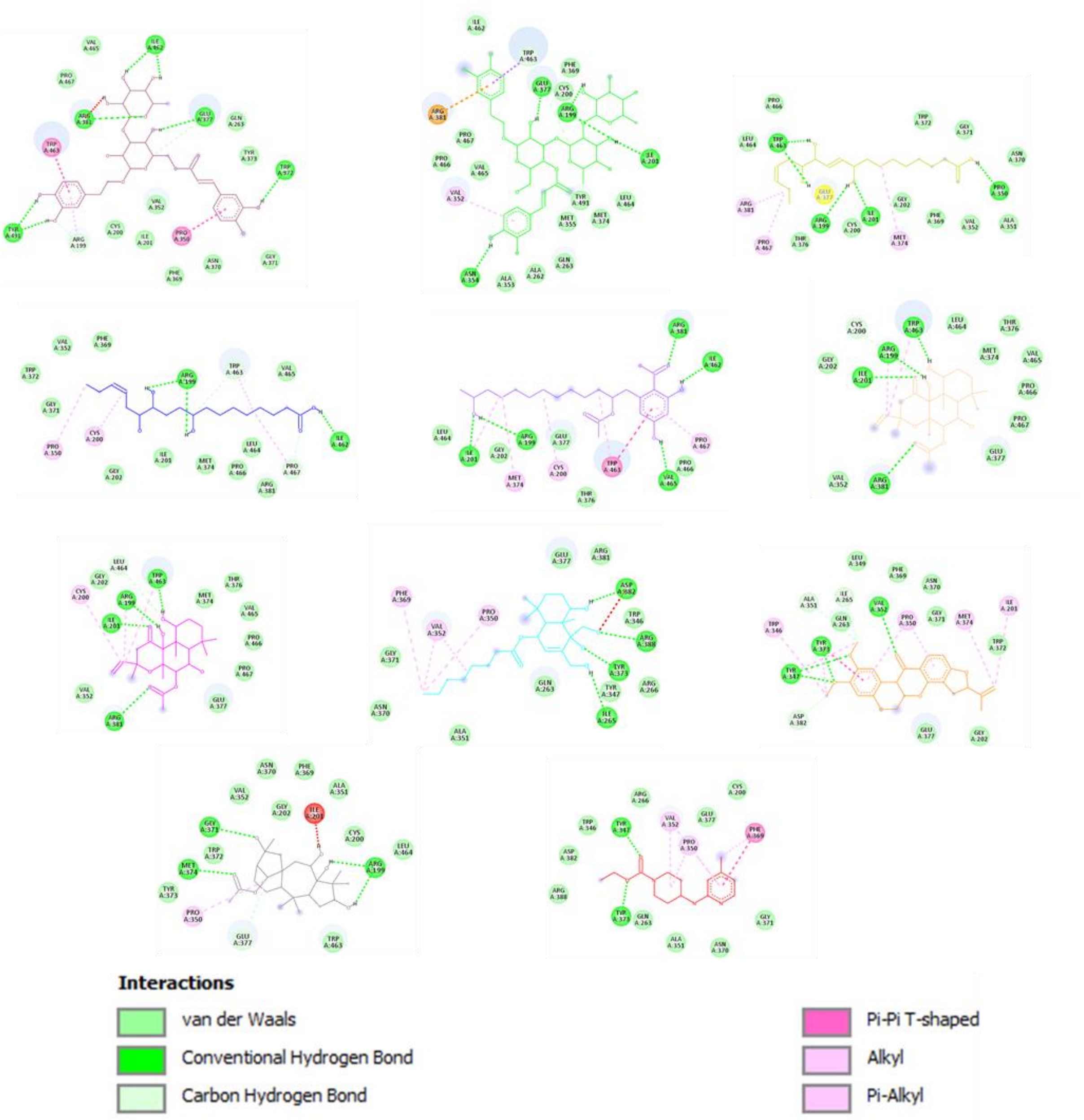

Figure S7. Interactions of compounds **55** (Raspberry), **57** (Green), **85** (Limon), **88** (Blue), **91** (Purple blue), **101** (Wheat), **106** (Magenta), **108** (Cyan), **109** (Orange), and **113** (Gray), with amino acid when they were docked into iNOS protein (**PDB ID 3E7G**).

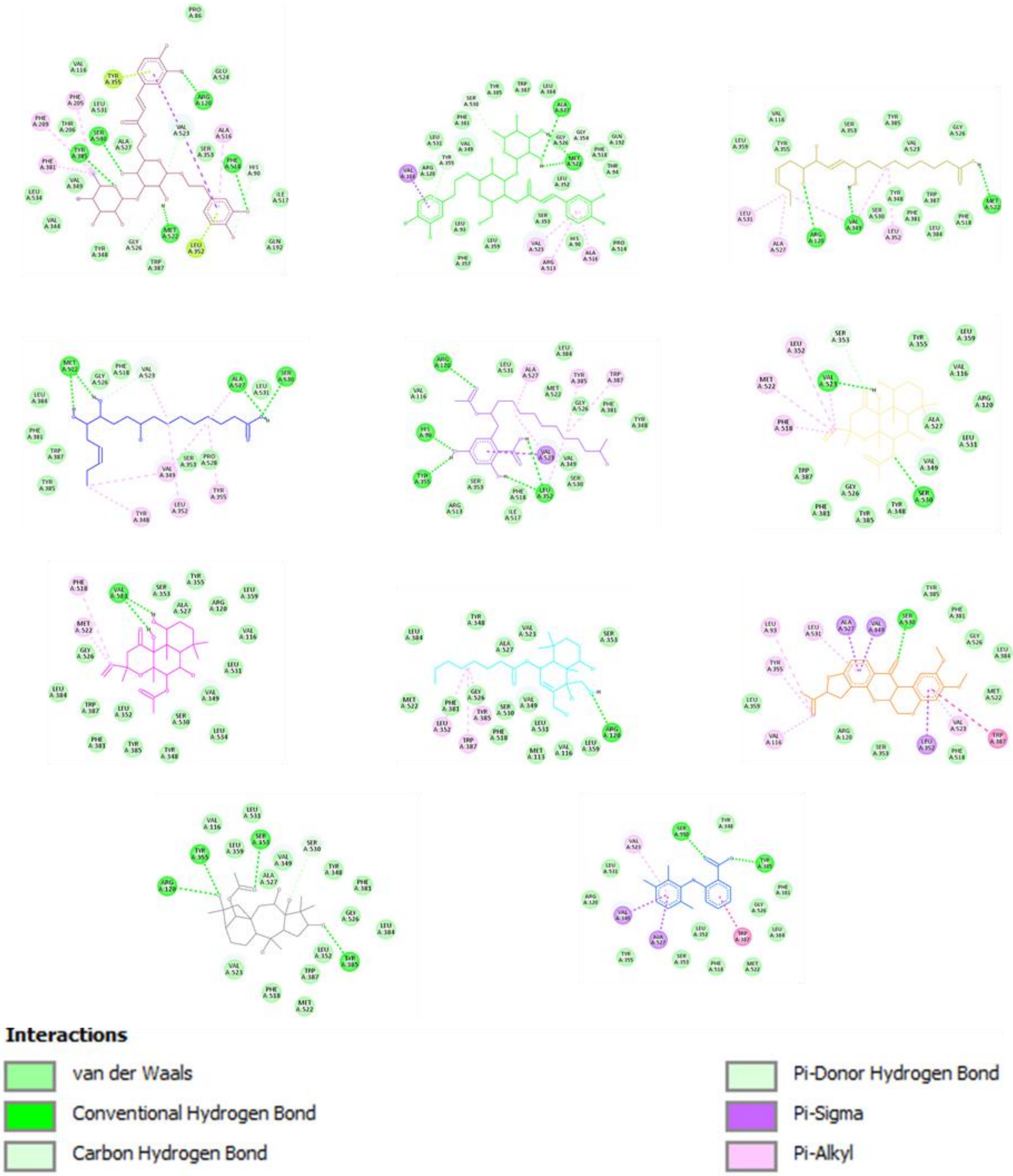

Figure S8. Interactions of compounds **55** (Raspberry), **57** (Green), **85** (Limon), **88** (Blue), **91** (Purple blue), **101** (Wheat), **106** (Magenta), **108** (Cyan), **109** (Orange), and **113** (Gray), with amino acid when they were docked into COX-2 protein (**PDB ID 5IKQ**).

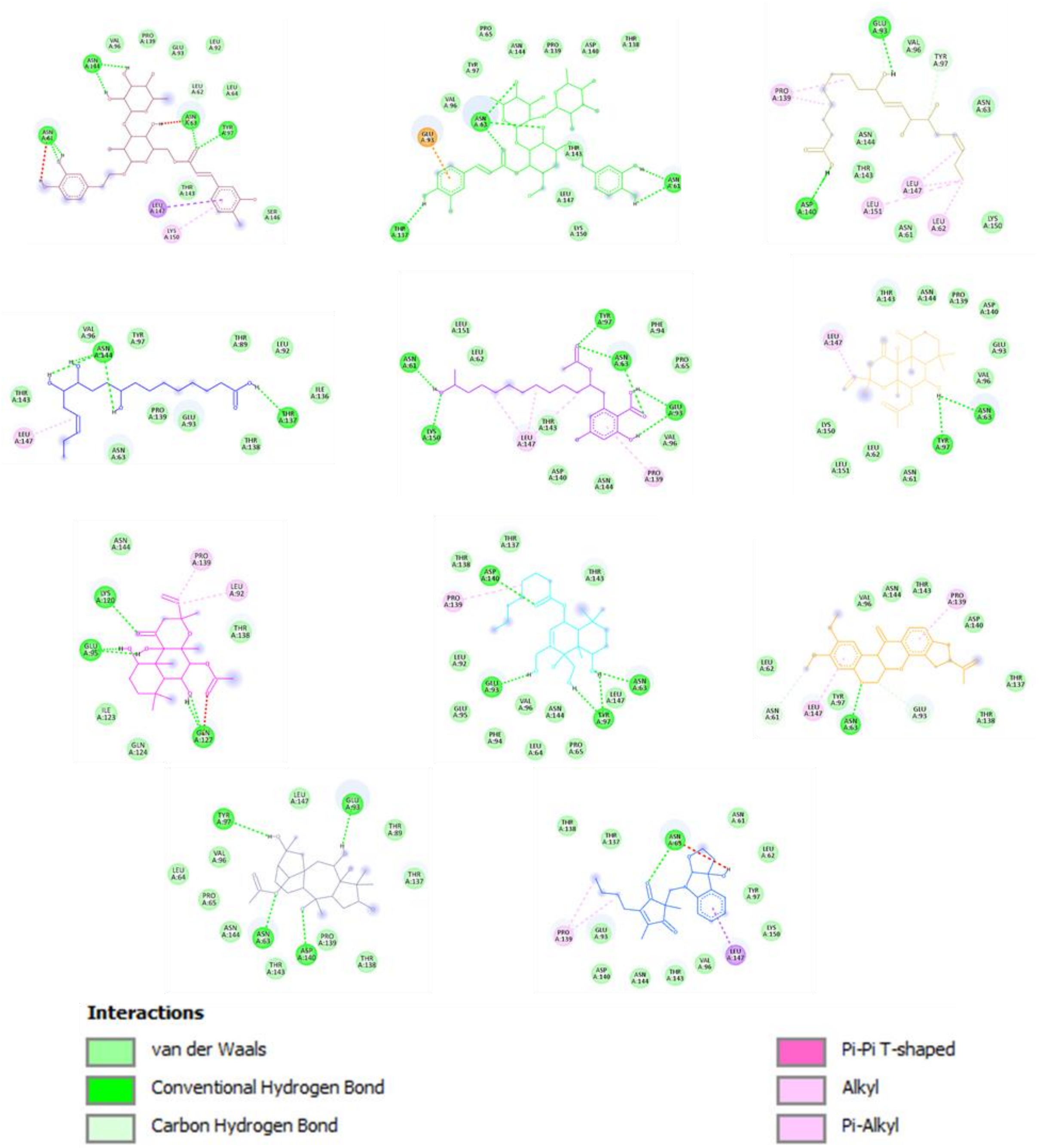

Figure S9. Interactions of compounds **55** (Raspberry), **57** (Green), **85** (Limon), **88** (Blue), **91** (Purple blue), **101** (Wheat), **106** (Magenta), **108** (Cyan), **109** (Orange), and **113** (Gray), with amino acid when they were docked into IL-6 protein (**PDB ID: 1ALU**).

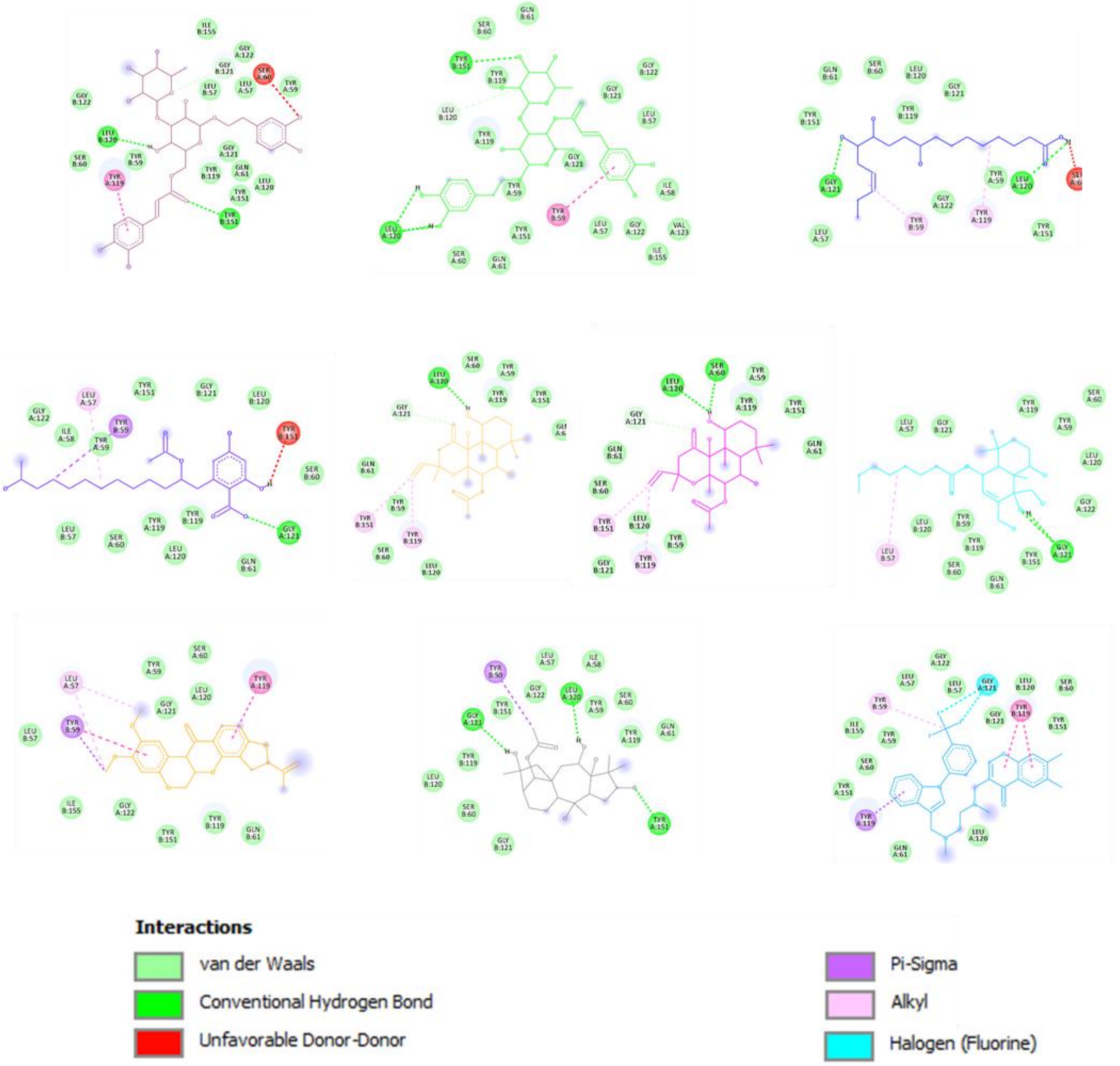

Figure S10. Interactions of compounds **55** (Raspberry), **57** (Green), **88** (Blue), **91** (Purple blue), **101** (Wheat), **106** (Magenta), **108** (Cyan), **109** (Orange), and **113** (Gray), with amino acid when they were docked into TNF-α protein (**PDB ID: 2AZ5**).

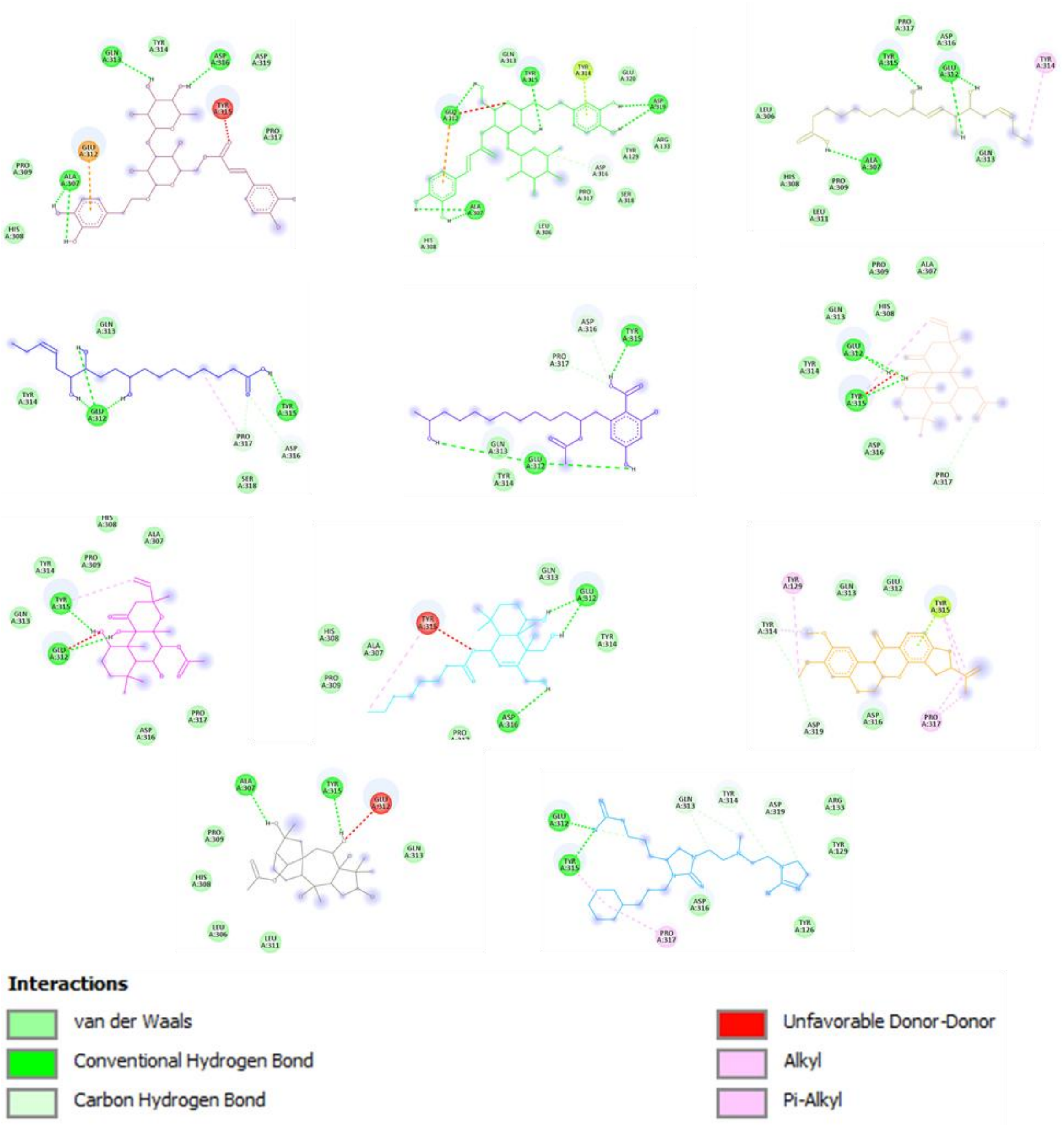

Figure S11. Interactions of compounds **55** (Raspberry), **57** (Green), **85** (Limon), **88** (Blue), **91** (Purple blue), **101** (Wheat), **106** (Magenta), **108** (Cyan), **109** (Orange), and **113** (Gray), with amino acid when they were docked into ERK protein (**PDB ID: 6NBS**).
